# Autophagy promotes organelle clearance and organized cell separation of living root cap cells in *Arabidopsis thaliana*

**DOI:** 10.1242/dev.200593

**Published:** 2022-06-16

**Authors:** Tatsuaki Goh, Kaoru Sakamoto, Pengfei Wang, Saki Kozono, Koki Ueno, Shunsuke Miyashima, Koichi Toyokura, Hidehiro Fukaki, Byung-Ho Kang, Keiji Nakajima

**Affiliations:** 1 Graduate School of Science and Technology, Nara Institute of Science and Technology, 8916-5 Takayama, Ikoma, Nara 630-0192, Japan; 2 School of Life Sciences, Centre for Cell and Developmental Biology and State Key Laboratory of Agrobiotechnology, The Chinese University of Hong Kong, Shatin, New Territories, Hong Kong, China; 3 Department of Biology, Graduate School of Science, Kobe University, Rokkodai, Kobe 657-8501, Japan

**Keywords:** *Arabidopsis thaliana*, Amyloplast, Autophagy, Cell separation, Root cap

## Abstract

The root cap is a multilayered tissue covering the tip of a plant root that directs root growth through its unique functions, such as gravity sensing and rhizosphere interaction. To maintain the structure and function of the root cap, its constituent cells are constantly turned over through balanced cell division and cell detachment in the inner and outer cell layers, respectively. Upon displacement toward the outermost layer, columella cells at the central root cap domain functionally transition from gravity-sensing cells to secretory cells, but the mechanisms underlying this drastic cell fate transition are largely unknown. Here, using live-cell tracking microscopy, we show that organelles in the outermost cell layer undergo dramatic rearrangements. This rearrangement depends, at least partially, on spatiotemporally regulated activation of autophagy. Notably, this root cap autophagy does not lead to immediate cell death, but is instead necessary for organized separation of living root cap cells, highlighting a previously undescribed role of developmentally regulated autophagy in plants.

This article has an associated ‘The people behind the papers’ interview.

## Abstract (precis)

**Summary:** Time-lapse microscopy reveals the spatiotemporal dynamics of intracellular reorganization associated with the functional transition and cell separation in *Arabidopsis* root caps, and the roles of autophagy in these processes.

## INTRODUCTION

**Fig. 1.**
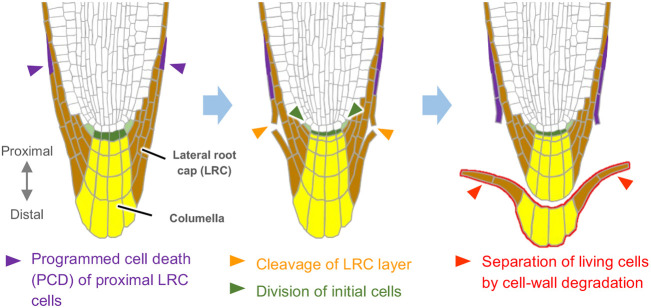
**Structure and cell detachment process of *Arabidopsis* root caps.** Key events constituting the cell separation sequence are marked by arrowheads. Proximodistal polarity as used in this study is defined on the left.

The root cap covers the tip of a plant root and protects the root meristem, where rapid cell division takes place to promote root elongation (Arnaud et al., 2010; Kumpf and Nowack, 2015). The root cap is also responsible for a number of physiological functions, such as gravity sensing to redirect the root growth axis (Strohm et al., 2012) and metabolite secretion for lubrication and rhizosphere interactions (Cannesan et al., 2012; Driouich et al., 2013; Hawes et al., 2016; Maeda et al., 2019). In addition to its unique functions, the root cap exhibits a striking developmental feature, namely, continuous turnover of its constituent cells (Fig. 1) (Kamiya et al., 2016). This cell turnover is enabled by the concerted production and detachment of cells at the inner stem cell layer and the outer mature cell layer, respectively. The outermost root cap cells detach from the root tip and disperse into the rhizosphere, creating a unique environment at the border between the root and the soil. The detaching root cap cells are called ‘border cells’ (Hawes and Lin, 1990). Cell turnover is common in animals but rarely occurs in plants, in which morphogenesis relies not only on the production of new cells, but also on the accumulation of mature and sometimes dead cells. Thus, the root cap is a unique experimental material in which to study how plant cells dynamically change their morphology and functions during tissue maintenance.

In the model angiosperm *Arabidopsis thaliana* (*Arabidopsis*), the root cap is composed of two radially organized domains: the central columella and the surrounding lateral root cap (LRC). Together, these domains constitute five-to-six cell layers along the root proximodistal axis (Fig. 1) (Dolan et al., 1993). In *Arabidopsis*, the outermost root cap cells do not detach individually, but instead separate as a cell layer (Fig. 1) (Driouich et al., 2007; Kamiya et al., 2016; Vicre et al., 2005). Previous studies revealed that detachment of *Arabidopsis* root cap cells is initiated by localized activation of programmed cell death (PCD) at the proximal LRC region and requires the functions of the NAC-type transcription factor SOMBRERO (SMB), a master regulator of root cap cell maturation (Bennett et al., 2010; Fendrych et al., 2014; Willemsen et al., 2008; Xuan et al., 2016). Although SMB is expressed in all root cap cells and acts as a master regulator of cell maturation in the root cap, two related NAC-type transcription factors, BEARSKIN 1 (BRN1) and BRN2, are specifically expressed in the outer two cell layers of the root cap (Bennett et al., 2010; Kamiya et al., 2016). BRN1 and BRN2 share high sequence similarities and redundantly promote the separation of central columella cells. Cell separation in plants requires partial degradation of cell walls. Indeed, *ROOT CAP POLYGALACTURONASE* (*RCPG*), encoding a putative pectin-degrading enzyme, acts downstream of *BRN1* and *BRN2*, and BRN1 can directly bind to the *RCPG* promoter (Kamiya et al., 2016). *CELLULASE 5* (*CEL5*), encoding a putative cellulose-degrading enzyme, is also implicated in cell separation in the root cap (del Campillo et al., 2004).

Previous electron microscopy studies reported profound differences in the intracellular organization between the inner and outer root cap cells of *Arabidopsis* (Maeda et al., 2019; Sack and Kiss, 1989). As expected from their gravity-sensing functions, columella cells in the inner layers accumulate large amyloplasts. Amyloplasts are specialized plastids containing starch granules and act as statoliths in the gravity-sensing cells (statocytes) of both roots and shoots (Nakamura et al., 2019). By contrast, fully maturated columella cells at the outermost root cap layer do not contain large amyloplasts, but instead accumulate secretory vesicles (Maeda et al., 2019; Poulsen et al., 2008). Thus, the observed difference in subcellular structures corresponds well with the functional transition of columella cells from gravity-sensing cells to secretory cells (Blancaflor et al., 1998; Maeda et al., 2019; Vicre et al., 2005). Before detachment, the outermost root cap cells contain a large central vacuole, likely for the storage of various metabolites (Baetz and Martinoia, 2014). In addition, a novel role of cell death promotion has been proposed for the large central vacuole in LRC cells (Fendrych et al., 2014).

In eukaryotes, dispensable or damaged proteins and organelles are degraded by a self-digestion process called autophagy (Mizushima and Komatsu, 2011). Autophagy initiates with expansion of isolated membranes, which subsequently form spherical structures, called autophagosomes, and engulf target components. In later steps of this process, autophagosomes fuse with vacuoles, and the contents of autophagosomes is degraded by hydrolytic enzymes stored in the vacuole. When eukaryotic cells are subjected to stress conditions, such as nutrient starvation, autophagy is activated to recycle nutrients and maintain intracellular environments to sustain the life of cells and/or individuals (Mizushima and Komatsu, 2011). Autophagy plays an important role not only in stress responses, but also in development and differentiation, because autophagy-deficient mutants are nonviable in a variety of model organisms, including yeast, nematodes, fruit flies and mice (Mizushima and Levine, 2010). Genes encoding central components of autophagy, the core autophagy-related (*ATG*) genes, are conserved in the *Arabidopsis* genome (Hanaoka et al., 2002; Liu and Bassham, 2012). However, under normal growth conditions, autophagy-deficient *Arabidopsis* mutants grow normally except for an early senecesence phenotype (Hanaoka et al., 2002; Yoshimoto et al., 2009). Thus, the roles of autophagy in plant growth and development remain largely unknown.

In this study, we used motion-tracking time-lapse imaging to reveal the morphological and temporal dynamics of intracellular rearrangements that enable the functional transition of root cap cells in *Arabidopsis*. We established that autophagy-deficient *Arabidopsis* mutants are defective in cell clearance and vacuolization of the outermost root cap cells. Unexpectedly, the autophagy-deficient mutants were impaired in the organized separation of the outermost root cap layer. Thus, our study reveals a novel role of developmentally regulated autophagy in root cap differentiation and function.

## RESULTS

### Outermost columella cells undergo rapid organelle rearrangement before cell detachment

Whereas previous electron microscopy studies revealed profound differences in the intracellular structures between the inner and outer root cap cells (Maeda et al., 2019; Poulsen et al., 2008; Sack and Kiss, 1989), the spatiotemporal dynamics of subcellular reorganization in root cap cells have not been analyzed, because it is challenging to perform prolonged time-lapse imaging of the root tip, which quickly relocates as the root elongates. To overcome this obstacle, we developed a motion-tracking microscope system with a horizontal optical axis and a spinning-disc confocal unit. A similar system has been reported by another group (von Wangenheim et al., 2017). For long-term time-lapse imaging, we grew *Arabidopsis* roots between a block of agar medium and the bottom of the chamber slide at an ambient temperature (approximately 22°C) under continuous illumination except during image acquisition. This experimental set-up enabled high-magnification time-lapse confocal imaging of the tip of vertically growing roots for up to 6 days, allowing visualization of the cellular and subcellular dynamics of root cap cells during three consecutive detachment events (Fig. S1).

**Fig. 2.**
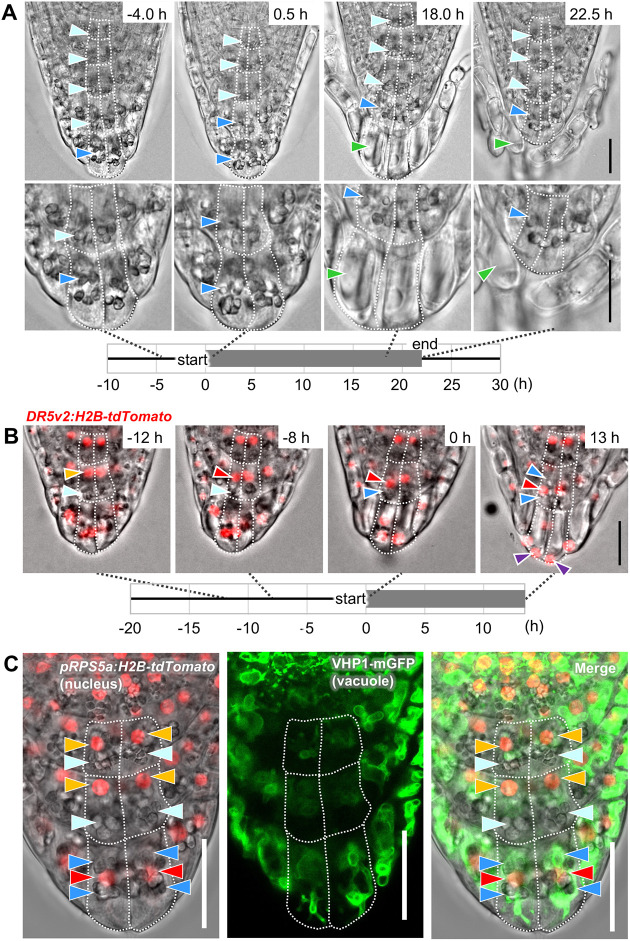
**Organelle rearrangement occurs in the outer root cap layers.** (A) Time-lapse images visualizing the sequences of root cap cell detachment and relocation of amyloplasts. Representative images before (−4.0 h), at the beginning (0.5 h) and near the end (18.0 and 22.5 h) of cell layer detachment are shown. Images in the bottom row are magnifications of the outer root cap layers shown in the upper row. Light-blue and dark-blue arrowheads indicate sedimenting and floating amyloplasts, respectively. The green arrowheads indicate highly vacuolated cells. A corresponding video is available as Movie 1. (B) Time-lapse images showing intracellular relocation of nuclei (red fluorescence of *DR5v2:H2B-tdTomato*) and amyloplasts (gray particles in the bright field). Purple arrowheads indicate nuclei localized at the distal pole of the cells. A corresponding video is available as Movie 2. (C) Confocal images visualizing differential localization of organelles between the inner and the outermost cell layers. In B,C, light-blue and dark-blue arrowheads indicate amyloplasts in the distal (bottom) and middle regions of the cell, respectively and orange and red arrowheads indicate red fluorescent nuclei localized in the proximal (upper) and middle regions of the cell, respectively. Green staining indicates vacuolar membranes. Images are representative of four (A and C) or three (B) roots, which all showed comparable patterns. The number of hours before or after the outermost layer began to detach in the proximal LRC region (defined as 0 h) is indicated in the upper-right corner of each image in A,B. The duration of cell detachment is indicated by the gray bar in the timeframe in A,B. Cell outlines are delineated by white-dashed lines. Scale bars: 20 µm.

Under our experimental conditions, the outermost root cap layer of wild-type *Arabidopsis* sloughed off at a largely fixed interval of approximately 38 h (Fig. S1F). This periodicity is comparable to that reported for roots growing on agar plates (Shi et al., 2018), indicating that our microscope system did not affect the cell turnover rate of the root cap. Bright-field observation confirmed that cell detachment initiated in the proximal LRC region and extended toward the central columella region (Fig. 1; Fig. S1A-D) (Fendrych et al., 2014; Shi et al., 2018). In concert with the periodic detachment of the outermost layer, the subcellular structures of the neighboring inner cell layer (hereafter called the second outermost layer) rearranged dynamically (Fig. 2A; Movie 1). Before detachment of the outermost layer, columella cells in the inner three-to-four cell layers contained large amyloplasts that sedimented toward the distal (bottom) side of the cell (Fig. 2A, −4.0 h, light-blue arrowheads), whereas those in the outermost layer were localized in the middle region of the cell (Fig. 2A, −4.0 h, dark-blue arrowhead). Soon after the outermost layer started to detach at the proximal LRC region, the amyloplasts in the second outermost layer relocated toward the middle region of the cell, resulting in a localization pattern similar to that of the outermost layer (Fig. 2A, 0.5 h, dark-blue arrowheads). Toward the completion of the cell separation, rapid vacuolization and shrinkage of amyloplasts took place in the outermost layer and the detached cells were fully vacuolated (Fig. 2A, 18.0 h and 22.5 h, green arrowhead).

Using plants expressing nuclear-localized red fluorescent proteins (*DR5v2:H2B-tdTomato*), we visualized the dynamic relocation of nuclei in the outer root cap layers, as well as the temporal relationship of this relocation with amyloplast movement (Fig. 2B; Movie 2). In the second outermost layer, nuclei relocated from the proximal (upper) to middle region of each cell a few hours before the neighboring outermost layer started to detach (Fig. 2B, −8 h, red arrowhead). This nuclear migration was followed by the relocation of amyloplasts around the time when the neighboring outermost layer initiated detachment at the proximal LRC region (Fig. 2B, 0 h, dark-blue arrowhead). In later stages, the amyloplasts surrounded the centrally localized nucleus (Fig. 2B, 13 h, dark-blue arrowhead). In the outermost cells, nuclei migrated further to localize to the distal pole of the cell (Fig. 2B, 13 h, purple arrowheads).

We also visualized dynamic changes in vacuolar morphology using plants expressing a tonoplast marker (*VHP1-mGFP*) (Segami et al., 2014) (Fig. S2; Movie 3). Vacuoles in the inner columella cells were small and spherical, whereas those in the outer cells were large and tubular (Fig. S2, 5-23 h). Notably, in the outermost layer, vacuoles were dramatically enlarged and eventually occupied most of the volume of detaching root cap cells (Fig. S2, 35-47 h). Confocal imaging of plants expressing both tonoplast and nuclear markers (*VHP1-mGFP* and *pRPS5a:H2B-tdTomato*) (Adachi et al., 2011; Segami et al., 2014) revealed that both nuclei and amyloplasts were embedded in the meshwork of vacuolar membranes in the outermost cell layer, whereas, in the inner cell layer, amyloplasts were localized in a space devoid of vacuolar membranes (Fig. 2C). Taken together, our time-lapse microscopy imaging revealed a highly organized sequence of organelle rearrangement in the outer root cap cells as well as its close association with cell position and cell detachment.

### Autophagy is activated in the outermost root cap cells before their detachment

Autophagy is an evolutionarily conserved self-digestion system in eukaryotes that operates by transporting cytosolic components and organelles to the vacuole for nutrient recycling and homeostatic control (Mizushima and Komatsu, 2011). The rapid disappearance of amyloplasts and the formation of large vacuoles in the outermost root cap cells led us to hypothesize that autophagy underlies their dynamic subcellular rearrangements before cell detachment. To test this hypothesis, we examined whether autophagosomes, spherical membrane structures characteristic of autophagy, are formed in the root cap cells at the time and space corresponding to organelle rearrangement.

**Fig. 3.**
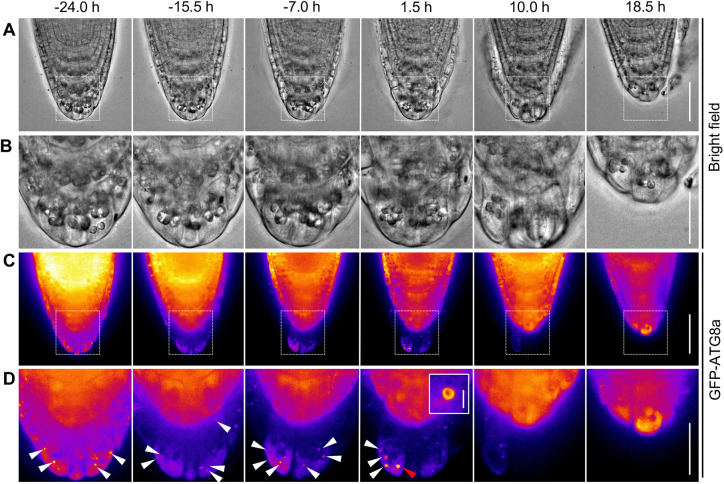
**Autophagosomes are formed specifically in the outermost root cap layer.** Representative bright-field (A,B) and GFP-ATG8a fluorescence (C,D) confocal time-lapse images of a *35Spro:GFP- ATG8a* root. Images in B and D are magnified images of the boxed regions in A and C, respectively. White arrowheads in D indicate autophagosomes marked by GFP-ATG8a. The signal is donut shaped, as is typical of autophagosome images in the later phase of detachment (red arrowhead at 1.5 h; inset shows an enlarged view). The time given above each column is relative to the point at which the outermost layer began to detach in the proximal LRC region (defined as 0 h). Images are representative of three roots, which all showed comparable patterns. A corresponding video is available as Movie 4. Scale bars: 50 µm in A,C; 20 µm in C,D; 2 µm in D (inset).

We first used an autophagosome marker, *35Spro:GFP-ATG8a*, which ubiquitously expresses GFP-tagged *Arabidopsis* ATG8a proteins, one of nine ATG8 proteins encoded by the *Arabidopsis* genome (Yoshimoto et al., 2004). ATG8 is a ubiquitin-like protein that, upon autophagy activation, is incorporated into the autophagosome membranes as a conjugate with phosphatidylethanolamine (Liu and Bassham, 2012). Our time-lapse confocal imaging revealed uniform localization of GFP-ATG8a fluorescence in the inner cell layers, suggesting that autophagic activity was low in these cells (Fig. 3; Movie 4). By contrast, in detaching outermost cells, dot-like signals of GFP-ATG8a became evident and increased in number and size (Fig. 3D, −24.0-1.5 h). During later stages of detachment, the GFP-ATG8a signal largely disappeared in the outermost cells before their detachment (Fig. 3D, 10.0 h). After the outermost cell layer had detached, the inner cells (i.e. the new outermost cells) still showed a uniform GFP-ATG8 signal (Fig. 3D, 18.5 h). During the later phase of cell detachment, the GFP-ATG8a signal exhibited ring-like shapes, which is typical of autophagosomes in confocal microscopy imaging (Fig. 3D, 1.5 h, red arrowhead and magnified image in the inset).

**Fig. 4.**
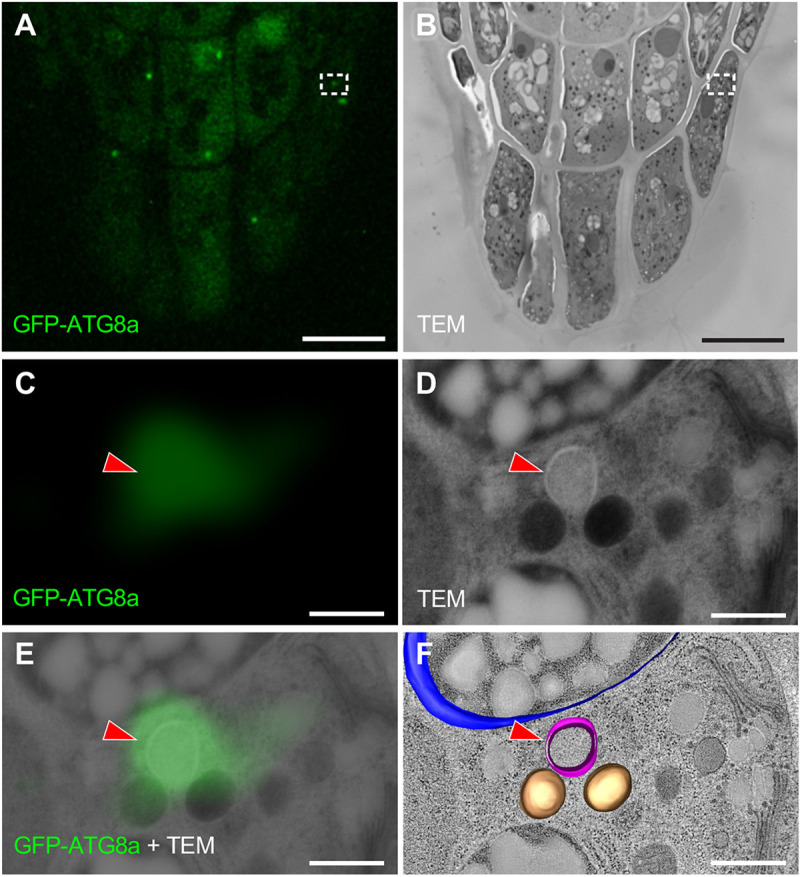
**CLEM imaging reveals localization of GFP-ATG8a in autophagosomes.** (A,B) GFP fluorescence (A) and TEM (B) images of a section from a *GFP-ATG8a* root cap. (C-E) Magnification of the regions boxed in A,B showing GFP-ATG8a fluorescence (C), TEM (D), and the merged image (E). Red arrowheads in C-F indicate an autophagosome with GFP-ATG8a fluorescence. (F) 3D electron tomographic model of an amyloplast (blue), two mitochondria (brown) and an autophagic compartment (magenta) overlaid with the TEM image. Scale bars: 10 µm in A,B; 500 nm in C-F.

To further establish whether the GFP-ATG8a-labeled puncta corresponded to the typical double membrane-bound autophagosome, we performed a correlative light and electron microscopy (CLEM) analysis (Fig. 4) (Wang and Kang, 2020). GFP fluorescence precisely colocalized with spherical structures typical of autophagosomes (Fig. 4C-F). Together, our observations confirmed that autophagy is activated in the outermost columella cells before their detachment.

### Autophagy promotes organelle rearrangement in the outermost root cap cells

**Fig. 5.**
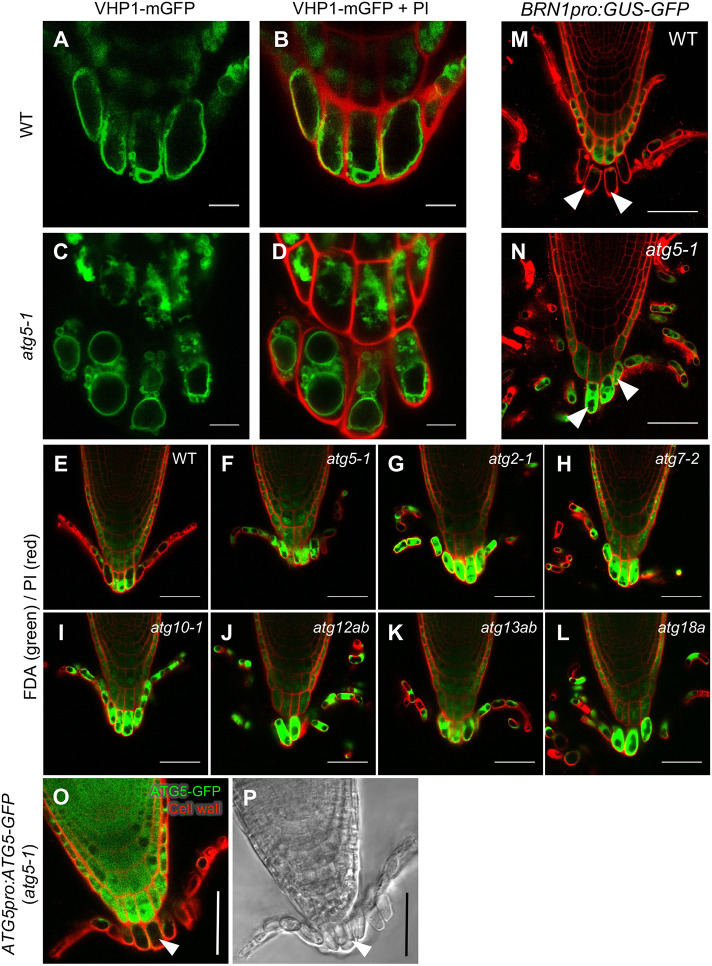
**Vacuolization and cytosol digestion are inhibited in detaching columella cells in *atg* mutants.** (A-D) Vacuolar morphologies in wild-type (A,B) and *atg5-1* (C,D) columella cells. (A,C) VHP1-mGFP fluorescence (green). (B,D) Merged images with PI-stained cell walls (red). (E-L) Retention of the cytosol in the detaching root cap cells of various *atg* mutants (F-L) compared with wild type (E). Cytosol and cell walls were stained with FDA (green) and PI (red), respectively. (M,N) Cytosolic GUS-GFP proteins expressed under the outer layer-specific *BRN1* promoter revealed cytosol digestion in the detaching root cap cells of the wild type but cytosol retention in the corresponding *atg5-1* cells (white arrowheads). (O,P) Vacuolization and cytosol digestion defects of detaching *atg5-1* root cap cells were complemented by the *ATG5-GFP* transgene (white arrowheads). Images are representative of three (A-N) or four (O,P) roots for each genotype, which all showed comparable patterns. Scale bars: 10 µm in A-D; 50 µm in E-P.

To examine whether autophagy plays a role in the maturation of columella cells, we phenotypically characterized autophagy-deficient mutants. *ATG* genes encoding autophagy components occur in the genomes of *Arabidopsis* and other model plant species (Hanaoka et al., 2002; Liu and Bassham, 2012). Among them, *ATG5* is a core *ATG* gene that is essential for autophagosome formation, similar to *ATG8*. In the loss-of-function *atg5-1* mutant (Yoshimoto et al., 2009), GFP-ATG8a signal was uniformly distributed throughout the cytosol both during and after cell detachment, indicating that autophagosome formation in detaching columella cells requires functional *ATG5* (Fig. S3; Movie 5). Furthermore, time-lapse observation revealed a loss of full vacuolation in the detaching outermost cells of *atg5-1* (Fig. S4A; Movie 6). In the detaching outermost cells of wild-type plants, a central vacuole enlarged to occupy most of the cell volume, whereas only a few spherical and small fragmented vacuoles were observed in the corresponding cells of *atg5-1* (Fig. 5A-D). The disappearance of iodine-stained large amyloplasts was not affected in the outer columella cells of *atg5-1* (Fig. S5A,B), and plastids in the *atg5-1* mutant exhibited abnormal morphologies dominated by tubular structures called stromules (Hanson and Hines, 2018), suggesting that autophagy plays a specific role in plastid restructuring and/or degradation (Fig. S5C,D). We also observed that the detaching *atg5-1* cells were strongly stained with fluorescein diacetate (FDA), a compound that emits green fluorescence when hydrolyzed in the cytosol, whereas fluorescence was limited to the cortical region of the corresponding wild-type cells (Fig. 5E,F). The cytosol of detaching columella cells also appeared to be retained in the FDA-stained roots of additional *atg* mutants, including *atg2-1*, *atg7-2*, *atg10-1*, *atg12ab*, *atg13ab* and *atg18a* (Fig. 5G-L), as well as in *atg5-1* plants expressing GUS-GFP fusion proteins under the outer layer-specific *BRN1* promoter (Fig. 5N, compare with Fig. 5M). Defects in vacuolization and cytosol digestion in *atg5-1* were complemented with an *ATG5-GFP* transgene, in which GFP-tagged ATG5 proteins were expressed under the *ATG5* promoter (Fig. 5O,P). Together, these observations demonstrate that autophagy plays a key role in cytosol digestion and vacuolization of detaching columella cells.

### Autophagy is required for the organized separation of the root cap cell layer

**Fig. 6.**
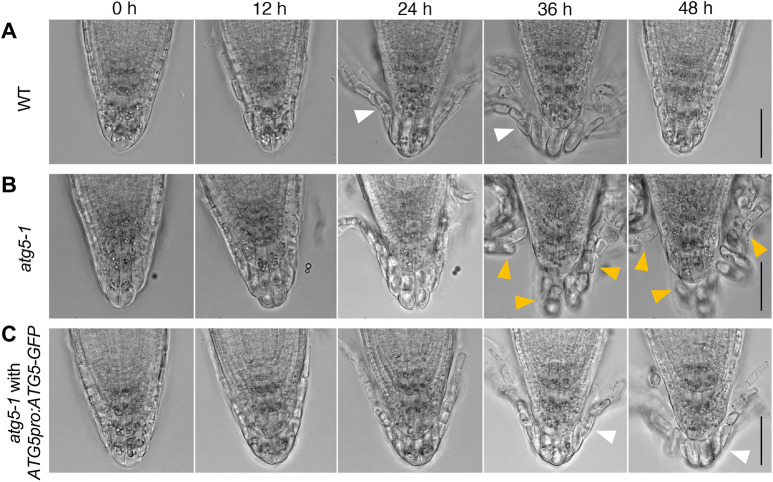
**Autophagy activation is required for organized separation of the outermost root cap cell layer.** (A-C) Time-lapse images of root cap detachment processes in wild-type (A), *atg5-1* (B) and *ATG5pro:ATG5-GFP atg5-1* (C) plants at the indicated time after the start of imaging*.* The outermost root cap cells detach as a layer (white arrowheads) in wild type (A) and *ATG5-GFP atg5-1* (C), whereas they detach individually in *atg5-1* (B, orange arrowheads). Images are representative of four roots for each genotype, which all showed comparable patterns. Corresponding videos are available as Movies 7-9. Scale bars: 50 µm.

In our time-lapse imaging analysis of *atg5-1*, the autophagy-deficient mutants exhibited cell detachment behavior that was distinct from that of the wild type. Whereas the outermost root cap cells detached as a cell layer in the wild type (Fig. 6A, white arrowheads; Movie 7) (Kamiya et al., 2016), those of *atg5-1* detached individually (Fig. 6B, orange arrowheads; Movie 8). This indicates that autophagy is required for not only organelle rearrangement, but also the organized separation of root cap cell layers, a behavior typically observed in the root cap of *Arabidopsis* and related species (Hamamoto et al., 2006; Hawes et al., 2002). The aberrant cell detachment behavior of *atg5-1* was complemented by the *ATG5-GFP* transgene (Fig. 6C, white arrowheads; Movie 9), confirming that the observed cell detachment defects were caused by the loss of *ATG5*.

**Fig. 7.**
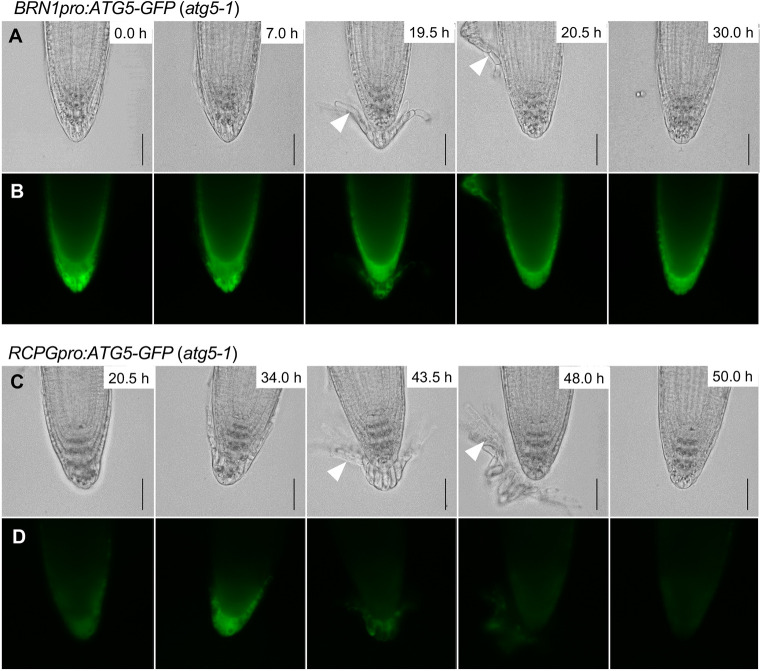
**Autophagy activation at the timing of cell wall degradation is sufficient for organized cell separation.** (A-D) Time-lapse images of root cap detachment processes in *BRN1pro:ATG5-GFP atg5-1* (A,B) and *RCPGpro:ATG5-GFP atg5-1* (C,D) plants at the indicated time after the start of imaging*.* The outermost root cap cells detach as a cell layer in both genotypes (white arrowheads) compared with the individual detachment observed for *atg5-1* (Fig. 6B). Bright-field (A,C) and GFP fluorescence (B,D) images are shown. Images are representative of four roots for each genotype, which all showed comparable patterns. Corresponding videos are available as Movies 10 and 11. Scale bars: 50 µm.

To clarify whether autophagy activation in the outermost cells is sufficient for organized cell separation, we established *atg5-1* plants expressing GFP-tagged ATG5 proteins under the *BRN1* and *RCPG* promoters, which drive transcription in the outer two cell layers and the outermost root cap layer, respectively (Kamiya et al., 2016). Time-lapse imaging revealed that organized separation of the outermost root cap cell layer was restored in both plant lines (Fig. 7A,B, white arrowheads; Movies 10 and 11). These observations, in particular the restoration of the layered cell separation by the *RCPG* promoter-driven ATG5-GFP, confirmed that autophagy activation in the detaching cells at the time of active cell wall degradation is sufficient for the organized separation of the outermost root cap layer.

## DISCUSSION

### Motion-tracking time-lapse imaging reveals rapid intracellular rearrangement associated with the functional transition of root cap cells

**Fig. 8.**
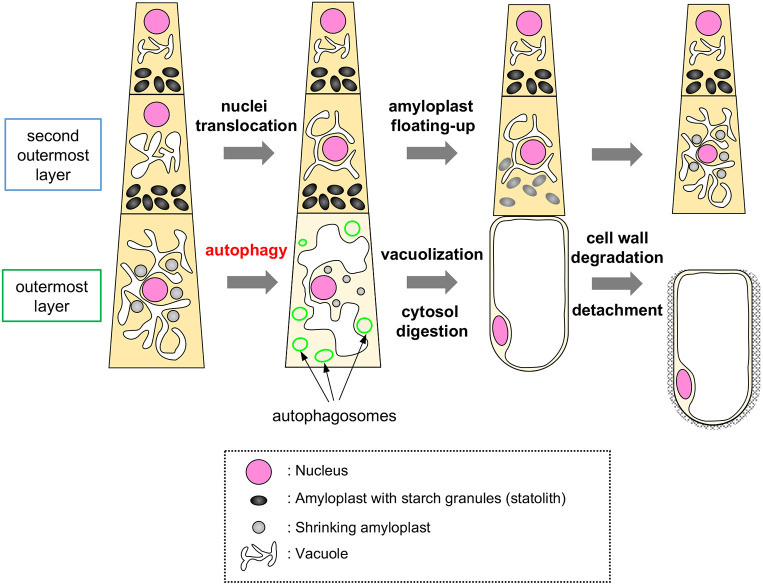
Schematic of the sequence of organelle rearrangement and autophagy activation during maturation and detachment of columella cells.

Cells constituting the root cap constantly turn over as a result of the balanced production and detachment of cells at the innermost and the outermost cell layers, respectively. During their lifetime, columella cells undergo a functional transition from being gravity-sensing statocytes to secretory cells as their position shifts (Blancaflor et al., 1998; Maeda et al., 2019; Sack and Kiss, 1989; Vicre et al., 2005). Whereas previous electron microscopy observations revealed a profound difference in the subcellular structures between the inner statocytes and outer secretory cells of the *Arabidopsis* root cap (Maeda et al., 2019; Moore and McClelen, 1983; Poulsen et al., 2008; Sack and Kiss, 1989), detailed temporal dynamics of organelle rearrangement in relation to the timing of cell displacement and detachment have not been analyzed. Our time-lapse observation using a motion-tracking microscope system with a horizontal optical axis clearly visualized both morphological and temporal details of organelle rearrangement in this transition, which occurred dynamically in the outermost cell layer in concert with the progression of cell separation (Fig. 8). Given that detached root cap cells are dispersed in the rhizosphere and act in plant defense through their secretory capacity (Driouich et al., 2013; Hawes et al., 2016), degradation of starch-containing amyloplasts and vacuolar expansion appear to be a reasonable differentiation trajectory in view of energy recycling and storage.

Here, a remaining question is what controls the spatiotemporal activation of this dramatic rearrangement of organelles in the root cap. The NAC-type transcription factors BRN1 and BRN2 are expressed specifically in the outer two cell layers of the root cap and are required for cell detachment (Bennett et al., 2010; Kamiya et al., 2016). This makes them good candidates for the upstream regulators of the organelle rearrangement. However, the outermost root cap cells of *brn1 brn2* mutants, although defective in cell detachment, are normally vacuolated and lack amyloplasts, similar to the wild type, indicating that at least a part of the organelle rearrangement is regulated independently of *BRN1* and *BRN2* (Bennett et al., 2010; Kamiya et al., 2016). However, our previous study suggested the existence of unknown positional cues that, together with another NAC-type transcription factor, SMB, promote the outer layer-specific expression of *BRN1* and *BRN2* (Kamiya et al., 2016). Future identification of factors transmitting such positional information could provide a clue as to the mechanism underlying position-dependent organelle rearrangement in the root cap.

### Autophagy is activated in the outermost root cap cells to promote cell clearance and vacuolization

Autophagosomes are double-membrane vesicles that engulf a wide range of intracellular components and transport them to vacuoles for degradation by lytic enzymes. The formation of autophagosomes and subsequent reduction of GFP-ATG8a signals support the occurrence of active autophagic flow and vacuolar degradation in the outermost root cap layer (Fig. 3). Such active autophagic transport may supply membrane components, thereby facilitating water influx into the vacuoles by increasing osmotic pressure, leading to enhanced vacuolization of the outermost root cap cells.

Whereas the autophagy-deficient *atg5-1* mutant could eliminate Lugol-stained amyloplasts from mature columella cells, similar to the wild type, the morphology of plastids in the detaching root cap cells was abnormal in *atg5-1*, which exhibited tubular structures typical of stromules (Fig. S3). Stromules arise from chloroplasts under starvation or senescence conditions. Under such stress conditions, the chloroplast contents are degraded via piecemeal-type organelle autophagy, in which stromules or chloroplast protrusions are believed to be engulfed by an autophagosome (Ishida et al., 2008), whereas damaged chloroplasts can be engulfed whole by an isolated membrane and transported into vacuoles (Izumi et al., 2013). Stromule formation in the autophagy-deficient *atg5-1* mutant suggests that amyloplast degradation in the outermost root cap cells proceeds in two steps: first, by autophagy-independent degradation of starch granules and stromule formation and, second, by piecemeal chloroplast autophagy. However, autophagy-dependent amyloplast degradation also occurs as part of the root hydrotropic response, in which some starch-containing amyloplasts are engulfed directly by autophagosome-like structures (Nakayama et al., 2012). Together, these observations suggest that multiple amyloplast degradation pathways exist in the *Arabidopsis* root cap, with different contributions by autophagy.

Although this study clearly demonstrates the role of autophagy in organelle rearrangement in the root cap, the spatiotemporal regulation of autophagy activation has yet to be investigated. Root cap autophagy appears to operate via a canonical macro-autophagy pathway mediated by the components encoded by *ATG* genes (Fig. 5) (Liu and Bassham, 2012). Autophagy is induced by various abiotic and biotic stresses, such as nutrient starvation. Under these conditions, SNF-RELATED KINASE 1 (SNRK1) and TARGET OF RAPAMYCIN (TOR) protein kinase complexes function as key regulators (Liu and Bassham, 2012; Mizushima and Komatsu, 2011). By contrast, root cap autophagy occurred in plants growing on a sterile nutrient-rich medium in our experiments, suggesting that root cap autophagy is activated independently of nutrient starvation and biotic stress. Instead, activation of root cap autophagy appears to be closely associated with the process of cell detachment, which, in turn, is known to be regulated by intrinsic developmental programs (Dubreuil et al., 2018; Shi et al., 2018). Again, *BRN1* and *BRN2* are unlikely to regulate root cap autophagy because cell clearance and vacuolization normally occur in the outermost root cap cells of *brn1 brn2* mutants.

### Autophagy is required for the organized separation of *Arabidopsis* root cap cells

Autophagy promotes organelle rearrangement associated with the differentiation of secretory cells that subsequently slough off to disperse into the rhizosphere. Based on this, we expected that the loss of autophagy would inhibit or delay cell detachment in the root cap. Somewhat unexpectedly, however, autophagy-deficient *atg5-1* mutants showed a phenotype suggestive of enhanced cell detachment (Fig. 6). In *Arabidopsis* and related species, the outermost root cap cells separate as a layer, rather than as isolated cells (Driouich et al., 2010, 2007; Kamiya et al., 2016). Although the physiological significance of this detachment behavior has not been demonstrated so far, it has been hypothetically linked with a capacity to secrete mucilage, a mixture of polysaccharides implicated in plant defense, aluminum chelating and lubrication (Driouich et al., 2010; Maeda et al., 2019).

Previous genetic studies suggested that cell wall pectins may regulate root cap cell detachment; when pectin-mediated cell-cell adhesion was compromised by mutations in genes encoding putative pectin-synthesizing enzymes or overexpression of *RCPG* encoding a root cap-specific putative pectin-hydrolyzing enzyme, root cap cells sloughed off as isolated cells (Driouich et al., 2010; Kamiya et al., 2016). Moreover, the morphology of detaching root cap cell layers was altered in the loss-of-function *rcpg* mutant, likely because of a failure in breaking cell-cell adhesion along the lateral cell edge of the separating cells (Kamiya et al., 2016). The similarity between the altered cell detachment behaviors between *atg5-1* and pectin-deficient plants suggests that autophagy functions in the control of cell wall integrity during root cap cell detachment. Both transport and modification of cell wall pectins require Golgi and Golgi-derived vesicles (Driouich et al., 2012; Wang et al., 2017). In outer root cap cells, small vesicles accumulate to perform their secretory functions (Driouich et al., 2013; Maeda et al., 2019; Wang et al., 2017). A mutation disrupting this secretory pathway results in the failure of root cap cell detachment (Poulsen et al., 2008). If autophagy is required for the timely attenuation of such vesicular transport during the cell detachment program, a lack of autophagy should lead to prolonged secretion of cell wall-modifying enzymes, such as RCPG, resulting in enhanced loosening of cell-cell adhesion. Indeed, we recognized broader gaps at the apoplastic junctions of the distal cell-cell adhesion points in *atg5-1* than in the wild type (Movies 7 and 8). Future studies comparing secretory dynamics of cell wall-modifying enzymes in various genetic backgrounds using our live-imaging system could elucidate the molecular mechanism controlling the cell detachment behaviors in the root cap and the role of autophagy.

In summary, our study revealed the role of spatiotemporally regulated autophagy in cell clearance and vacuolization in root cap differentiation as well as in cell detachment. Although autophagy is known to promote tracheary element differentiation in *Arabidopsis* and anther maturation in rice (*Oryza sativa*), the roles of autophagy in these instances are linked to PCD (Escamez et al., 2016; Kurusu and Kuchitsu, 2017). Considering that autophagy is required for the functional transition and detachment of living columella cells, our study revealed a previously undescribed role of developmentally regulated autophagy in plant development.

## MATERIALS AND METHODS

### Plant materials and growth conditions

*A. thaliana* L. Heynh (*Arabidopsis*) accession Col-0 was used as the wild type. The *Arabidopsis* T-DNA insertional lines *atg5-1* (SAIL_129_B07), *atg7-2* (GK-655B06), *atg2-1* (SALK_076727), *atg10-1* (SALK_084434), *atg12a* (SAIL_1287_A08), *atg12b* (SALK_003192), *atg13a* (GABI_761_A11), *atg13b* (GK-510F06) and *atg18a* (GK_651D08) were described previously (Doelling et al., 2002; Hanaoka et al., 2002; Izumi et al., 2013; Thompson et al., 2005; Yoshimoto et al., 2004, 2009), as were *35Spro:CT-GFP*, *RPS5apro:H2B-tdTomato* and *VHP1-mGFP* (Adachi et al., 2011; Köhler et al., 1997; Segami et al., 2014). Seeds were grown vertically on *Arabidopsis* nutrient solution supplemented with 1% (w/v) sucrose and 1% (w/v) agar under a 16 h light/8 h dark condition at 23°C.

### Generation of transgenic plants

For *ATG5pro:ATG5-GFP*, a 4.5 kb genomic fragment harboring the ATG5-coding region and the 5′-flanking region was amplified by PCR and cloned into the pAN19/GFP-NOSt vector, which contained the GFP-coding sequence and the *Rhizobium* (*Agrobacterium*) nopaline synthase terminator (NOS). The resulting *ATG5-GFP* fragment was then transferred into *pBIN41* to give *ATG5pro:ATG5-GFP/pBIN41*.

Layer-specific rescue constructs of *ATG5-GFP* were constructed by amplifying the *ATG5-GFP* fragment from *ATG5pro:ATG5-GFP/pBIN41* and inserting it into pDONR221 using Gateway technology. The *ATG5-GFP* fragment was then transferred into *pGWB501:BRN1pro* and *pGWB501:RCPGpro*, which contained the *BRN1* and *RCPG* promoters, respectively, flanking the Gateway cassette in pGWB501 (Nakagawa et al., 2007). The cytosolic marker *GUS-GFP* was similarly constructed by inserting a *GUS-GFP* fragment into pENTR D-TOPO and then by transferring the insert into *pGWB501:BRN1pro* to give *BRN1pro:GUS-GFP*.

For *DR5v2:H2B-tdTomato*, a *DR5v2* promoter fragment was amplified by PCR from the *DRv2n3GFP* construct (Liao et al., 2015) and inserted into pGWB501 using the In-Fusion technique to give *pGWB501:DR5v2*. The *H2B-tdTomato* fragment in pENTR was transferred into *pGWB501:DR5v2*. Integrity of the cloned genes was verified by DNA sequencing. Transformation of *Arabidopsis* plants was performed using the floral dip method with *Rhizobium tumefaciens* strain C58MP90.

### Microscopy

Time-lapse imaging of the root cap was performed using two microscope systems developed in the corresponding authors' laboratory, which can automatically track the tip of vertically growing roots. Briefly, an inverted microscope (ECLIPSE Ti-E and ECLIPSE Ti2-E, Nikon) was tilted by 90° to orient the sample stage vertically. The motorized stage was controlled by Nikon NIS-elements software with the ‘keep object in view’ plug-in to track the tip of growing roots automatically. Three-day-old seedlings were transferred to a chamber slide (Lab-Tek chambered coverglass, Thermo Fisher Scientific) and covered with a block of agar medium. Time-lapse imaging was performed at an ambient temperature (∼22-23°C). To allow plant growth on the microscope stage, the samples were continuously illuminated by LEDs except for during image acquisition (typically at 5 min intervals for root tip tracking, each taking about 5 s). Fluorescence images were typically captured at 30 min intervals.

Confocal laser scanning microscopy was conducted with a Nikon C2 confocal microscope. Roots were stained with 10 µg/ml propidium iodide (PI). FDA staining was performed by soaking the roots in a solution containing 2 μg/ml FDA.

Iodine staining was performed as described previously (Segami et al., 2018). Roots were fixed in 4% (w/v) paraformaldehyde in PBS for 30 min under a vacuum at room temperature. The fixed sample was washed twice for 1 min each in PBS and cleared with ClearSee (Kurihara et al., 2015). The samples were transferred to 10% (w/v) xylitol and 25% (w/v) urea to remove sodium deoxycholate and then stained in a solution containing 2 mM iodine (Wako), 10% (w/v) xylitol and 25% (w/v) urea.

CLEM analysis was performed as described previously (Wang and Kang, 2020; Wang et al., 2019). GFP-ATG8a seedlings were grown vertically under a 16 h light/8 h dark cycle at 22°C for 7 days. Root tip samples expressing GFP were cryofixed with an EM ICE high-pressure freezer (Leica Microsystems) and embedded in Lowicryl HM20 resin at −45°C. Transmission electron microscopy (TEM) sections of 150 nm thickness were collected on copper or gold slot grids coated with formvar and examined for GFP after staining the cell wall with Calcofluor White. The grids were post-stained and GFP-positive cells were imaged under an H-7650 TEM (Hitachi High-Tech) operated at 80 kV. For electron tomography, tilt series were collected with a TF-20 intermediate-voltage TEM (Thermo Fisher Scientific). Tomogram calculation and three-dimensional (3D) model preparation were performed with the 3dmod software package (bio3d.colorado.edu).

## Supplementary Material

Supplementary information

Reviewer comments
