# Supplementary material for: Autophagy promotes organelle clearance and organized cell separation of living root cap cells in Arabidopsis thaliana
Source: Development. 2022 Jun 16;149(11):dev200593. doi: 10.1242/dev.200593 (PMC9245187; doi:10.1242/dev.200593)
Supplement: Supplementary information [file develop-149-200593-s1.pdf]

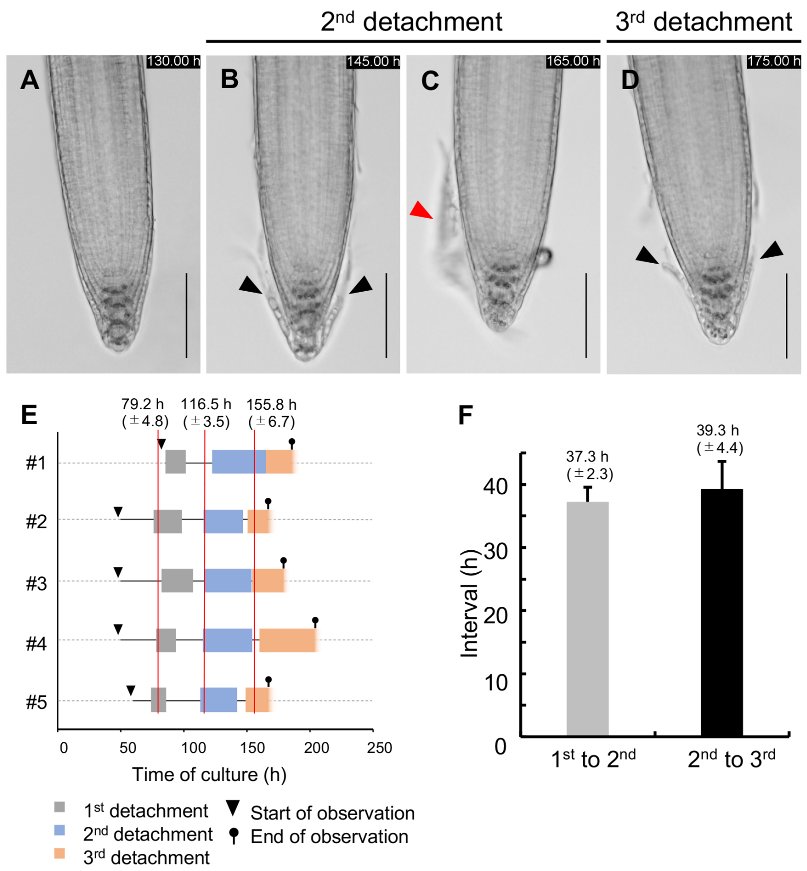

Fig. S1. Arabidopsis root cap cells detach at fixed intervals.

(A–D) Time-lapse images showing the periodic detachment of *Arabidopsis* root cap cells. Detachment of the outermost root cap layer initiates at the proximal LRC region and progressively extends toward the central columella region (B, black arrowheads). Detached root cap cells remain together as a single cell layer (C, red arrowhead). Detachment of the next cell layer initiates in the same manner as the previous one (D). Elapsed time after the start of culture is indicated in each panel. Scale bar, 100 µm.

(E) Schedule of the periodic detachment of root cap cell layers in five (#1–5) root samples each experiencing three rounds of root cap detachment. Gray, blue, and orange boxes indicate the duration from the start (initial detachment at the proximal LRC region) and the end (complete detachment at the columella region) of the first, second, and third cell layer, respectively. The x-axis indicates elapsed time (h) from the start of culture. Red lines indicate average time points of the start of detachment.

(F) Intervals between the start of detachment of the first and second cell layers (gray bar) and of the second and third cell layers (black bar). Mean and SE are shown ($n = 5$).

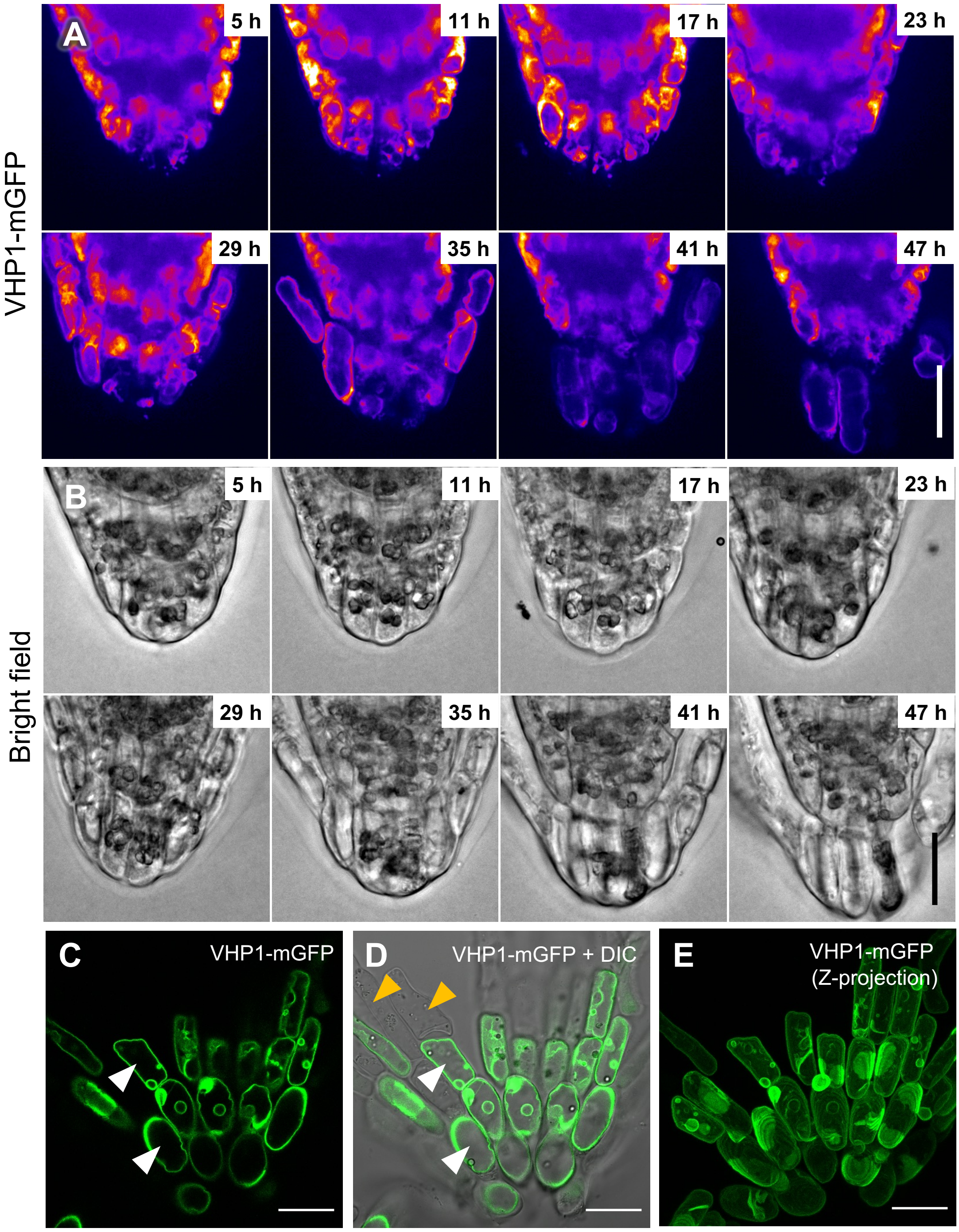
A
5 h
11 h
17 h
23 h
29 h
35 h
41 h
47 h
VHP1-mGFP
B
Bright field
C
VHP1-mGFP
D
VHP1-mGFP + DIC
E
VHP1-mGFP
(Z-projection)

Fig. S2. Vacuoles expand rapidly in the outermost root cap cells prior to their detachment.
(A, B) Time-lapse images showing vacuolar morphology based on tonoplast-localized VHP1-mGFP fluorescence (A) and bright-field images (B). In the outermost cells, vacuoles are initially small and fragmented (up to 17 h) and gradually expand to form large central vacuoles before cell detachment (41 h). Elapsed time after the start of observation is indicated in each panel. A corresponding video is available as Movie S3.
(C-E) Most of the cell volume was occupied by a large central vacuole in detaching root cap cells. Images of VHP1-mGFP fluorescence (C) and its overlay with a DIC image (D) are shown. A Z-stack projection encompassing a 50-µm depth is shown in (E). Note that cells at the center of the detached cell layer possess large central vacuoles as visualized by VHP1-mGFP (white arrowheads), whereas those at the periphery do not show fluorescence (orange arrowheads) likely due to the loss of cell viability.
Data are representative of three roots for each genotype, which all showed comparable patterns. Scale bar, 20 µm.

Development • Supplementary information

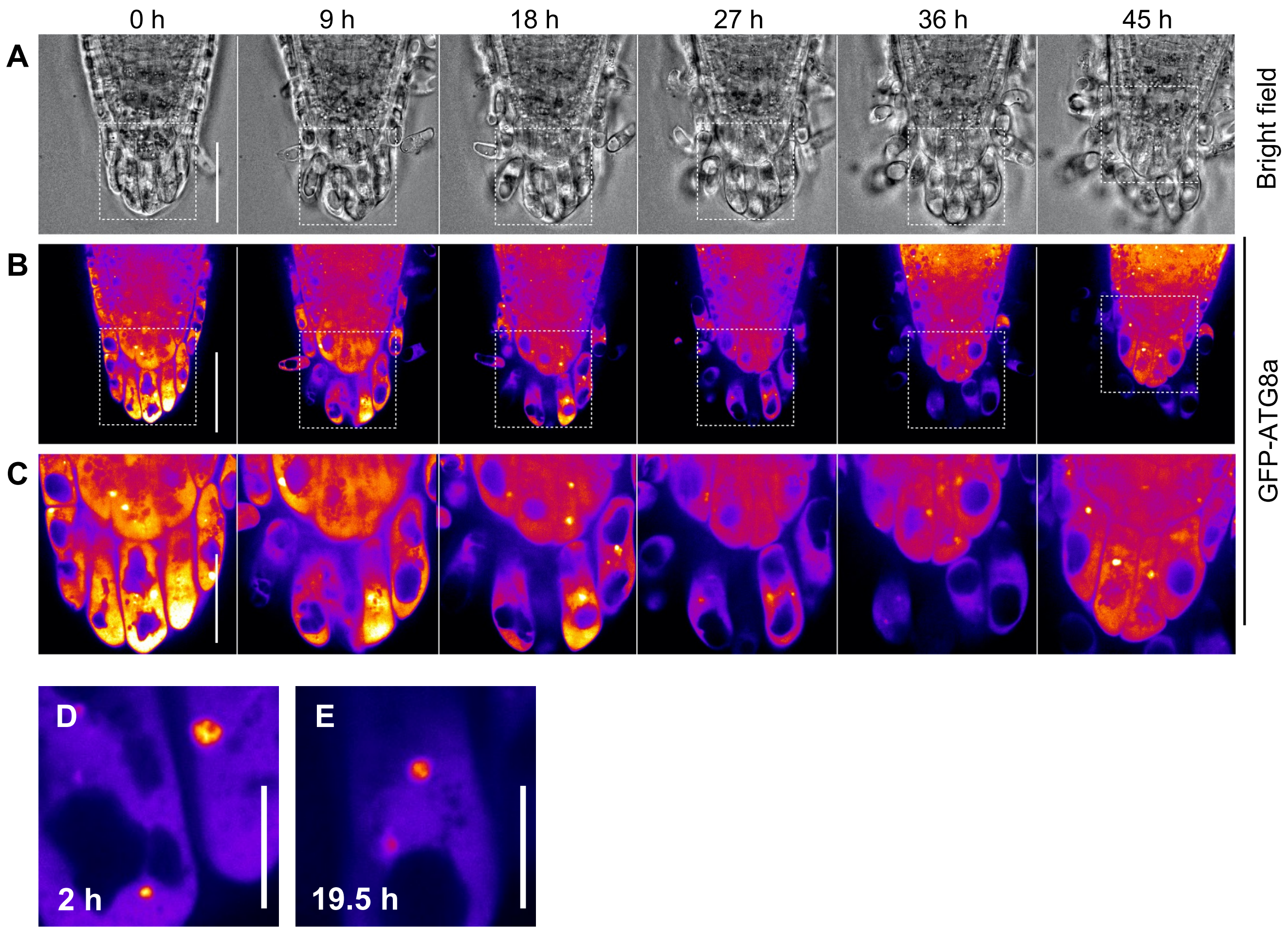

Fig. S3. Autophagosomes do not form in the detaching root cap cells of *atg5-1*. Time-lapse images of the *35Spro:GFP-ATG8a atg5-1* root tip. Bright-field (A) and GFP-ATG8a fluorescence images (B, C) are shown. Images in (C) are magnified views of boxed regions in (B) at respective time points. Note that the GFP-ATG8a signals were uniformly distributed throughout the cytosol. Occasionally, observed punctate signals did not form the donut shape typical of an autophagosome (D, E). Elapsed time after the start of observation is indicated at the top. Data are representative of three roots, which all showed comparable patterns. Scale bar, 50 µm (A, B); 20 µm (C); 10 µm (D, E). A corresponding video is available as Movie S5.

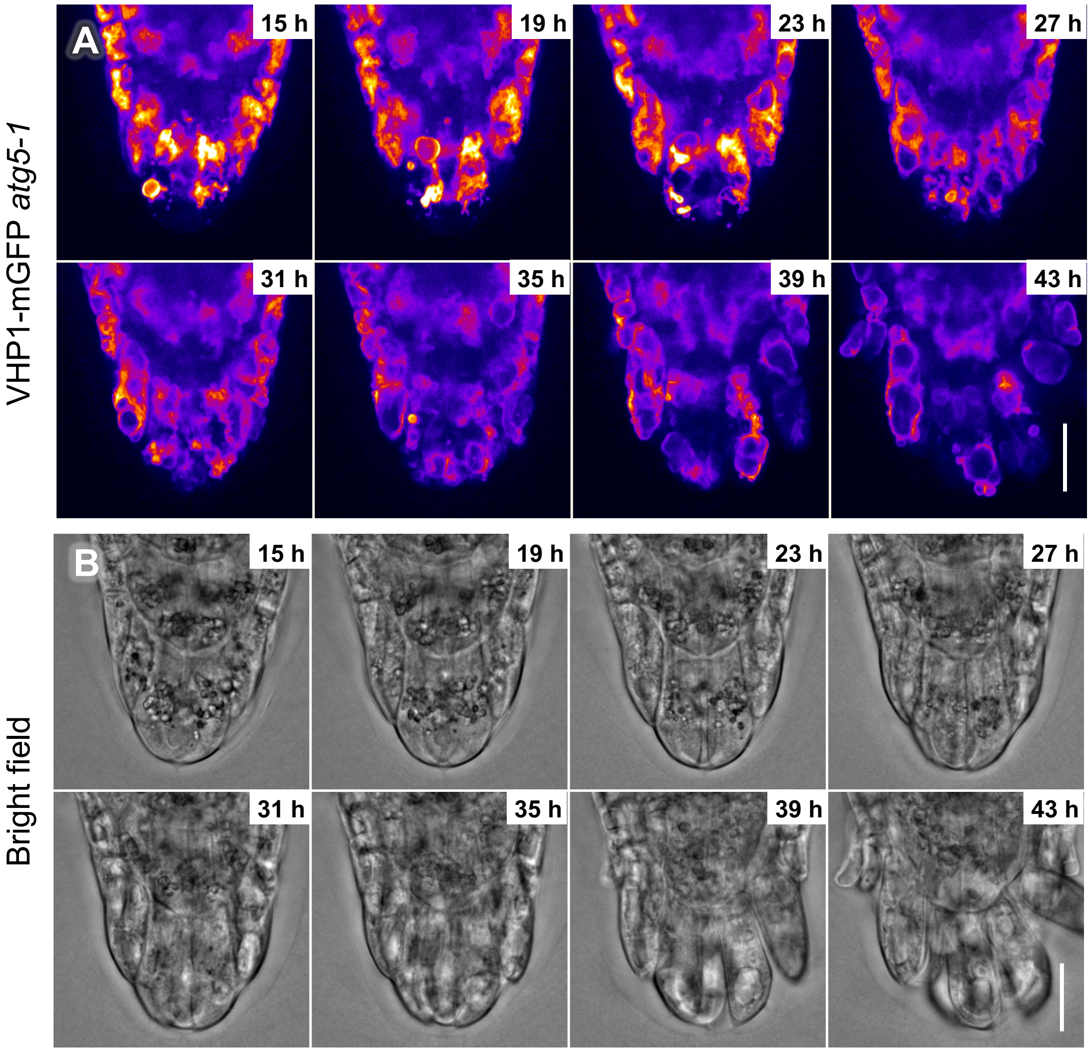

Fig. S4. Vacuolization and cytosol digestion do not occur in detaching *atg5-1* cells.

(A, B) Time-lapse images showing vacuolar morphology using tonoplast-localized VHP1-mGFP fluorescence (A) and corresponding bright-field images (B) in *atg5-1*. In the outermost cells, vacuoles are initially small and fragmented and gradually expand as those in the wild type but fail to expand fully (43 h). Elapsed time after the start of observation is indicated at the upper right corner of each panel. Data are representative of three roots, which all showed comparable patterns. A corresponding video is available as Movie S6. Scale bar, 20 µm (A, B).

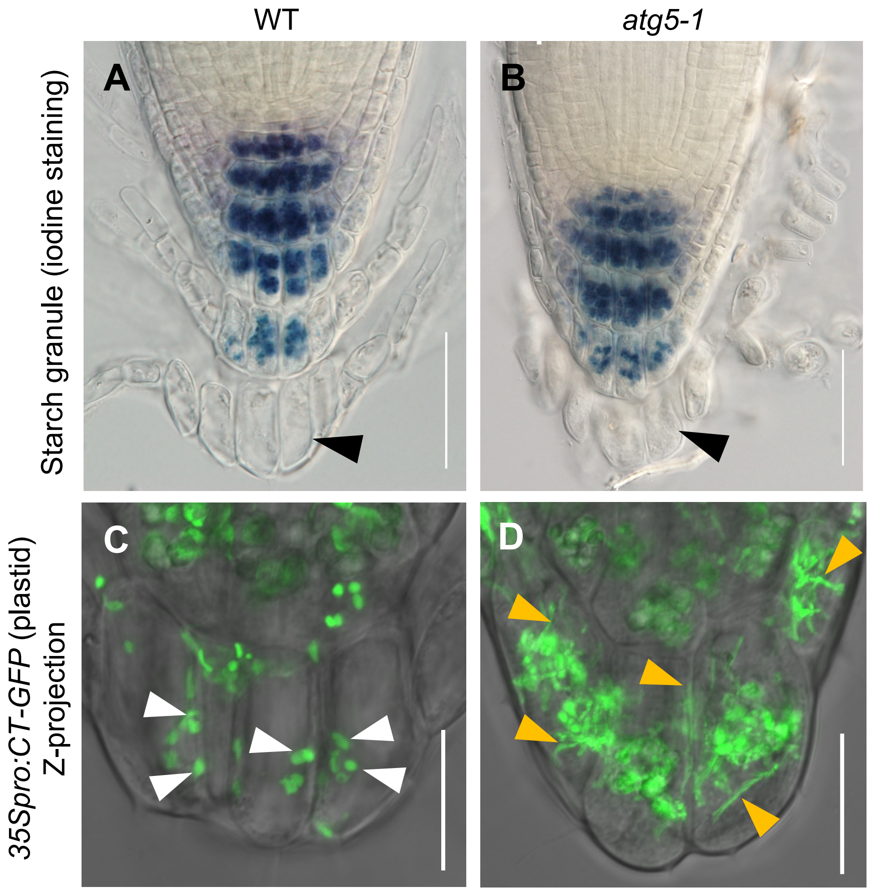

Fig. S5. Abnormal plastid morphology in *atg5-1* root cap cells.
(A, B) Amyloplasts in the outermost root cap cells lost starch granules in both the wild type (WT) and *atg5-1*. Black arrowheads indicate the detaching outermost cell layers.
(C, D) Amyloplasts exhibit abnormal morphologies in the outermost root cap cells of *atg5-1* (D) compared with those in the WT (C). Plastids are visualized by the CT-GFP fluorescence marker line. Note that small spherical plastids accumulate in the WT cells (white arrowheads), whereas those with tubular morphologies dominate in *atg5-1* cells (orange arrowheads).
Data are representative of three (A, B) or four (C, D) roots for each genotype, which all showed comparable patterns. Scale bar, 50 µm (A, B); 20 µm (C, D).

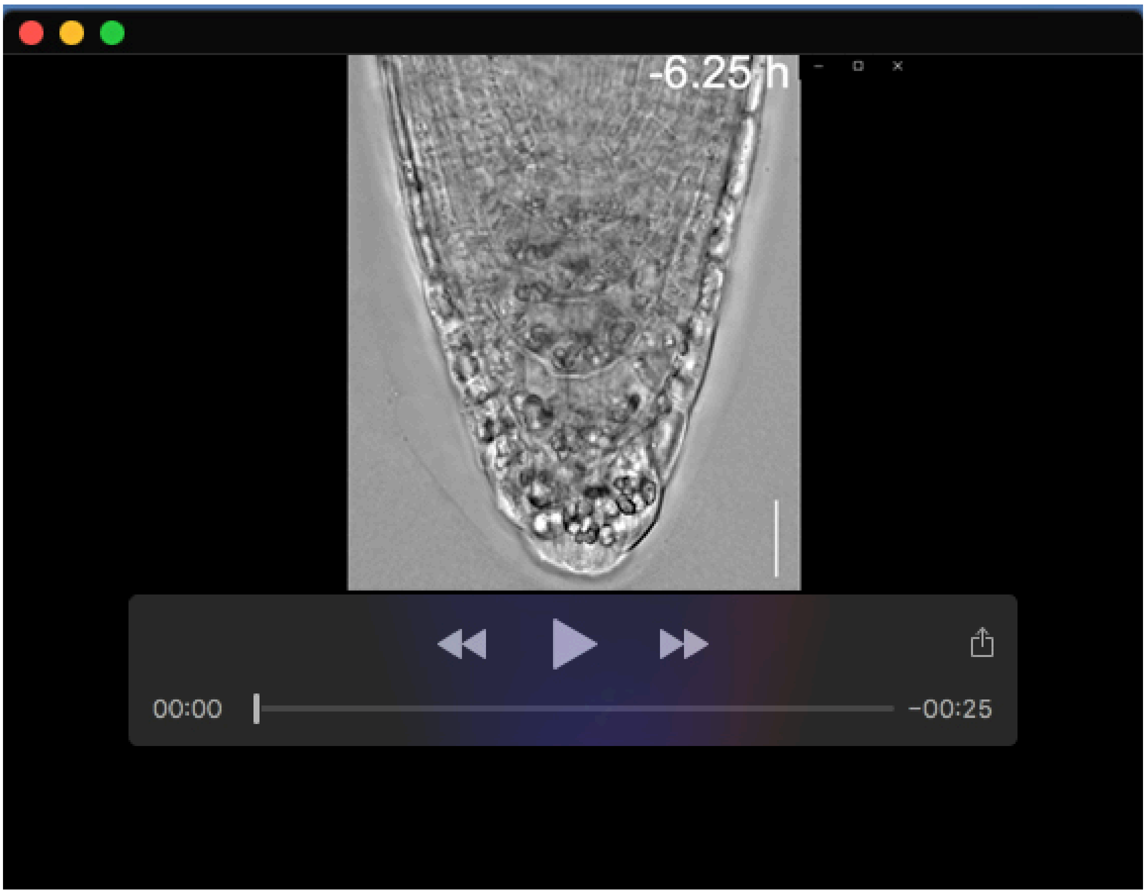

Movie 1. Time-lapse movie showing root cap cell detachment and organelle rearrangement in wild-type root cap cells Scale bar, 20 µm.

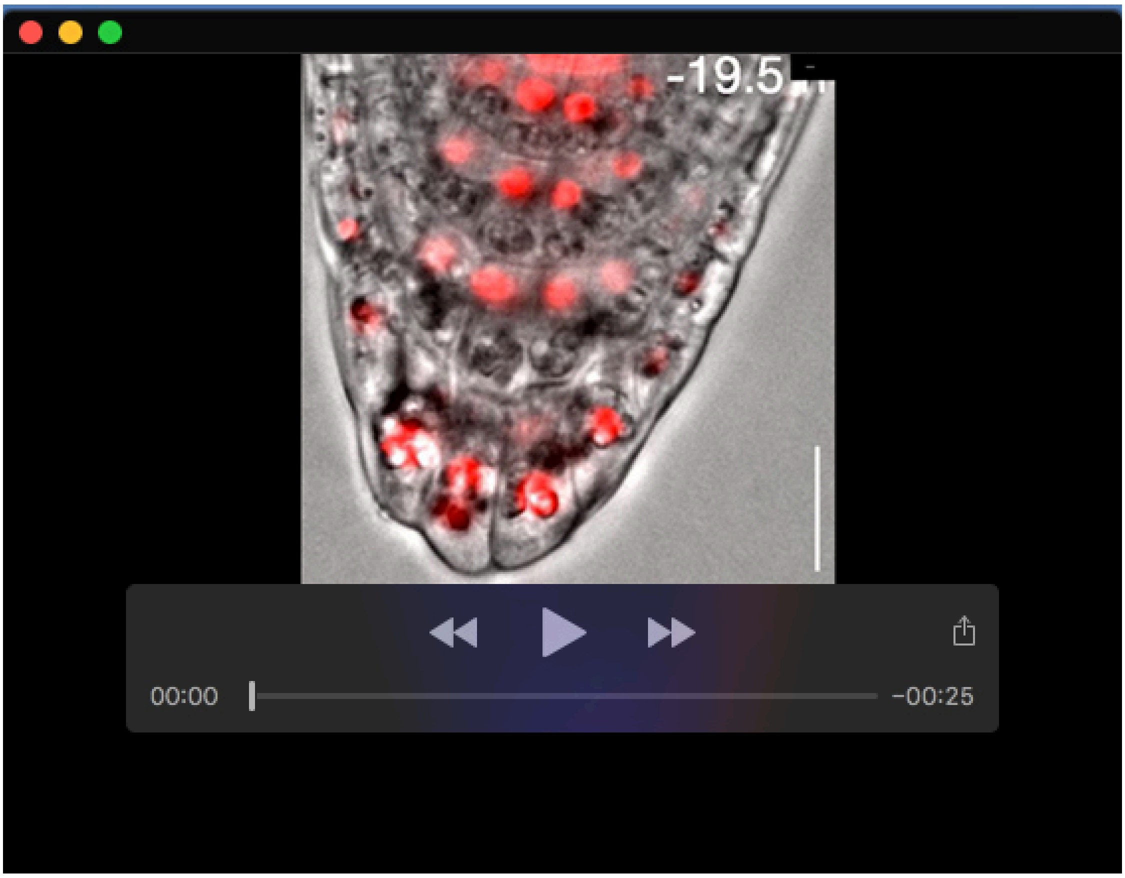

Movie 2. Time-lapse movie showing intracellular relocation of nuclei (red, *DR5v2:H2B-tdTomato*) and amyloplasts (gray particles in the bright field) in the root cap cells
Scale bar, 20 µm.

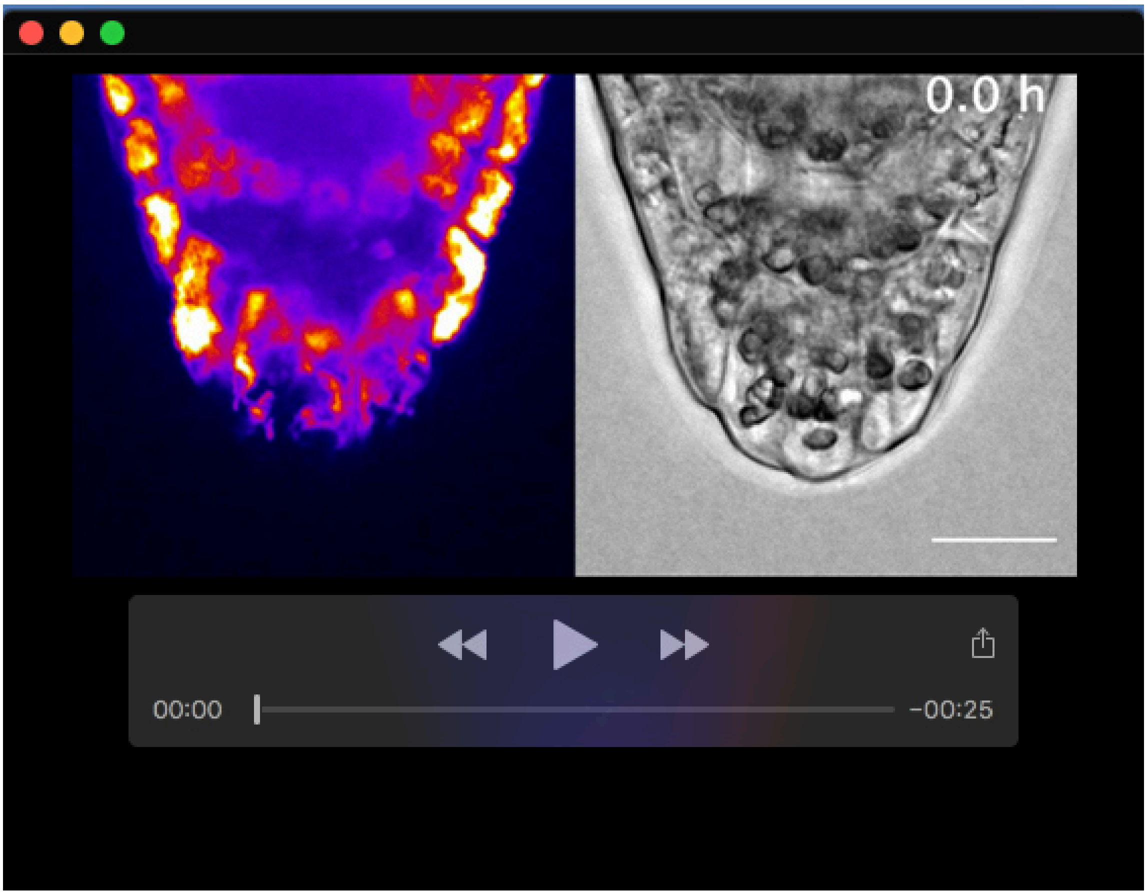

Movie 3. Time-lapse movie showing the morphological transition of vacuoles during cell detachment
Scale bar, 20 µm.

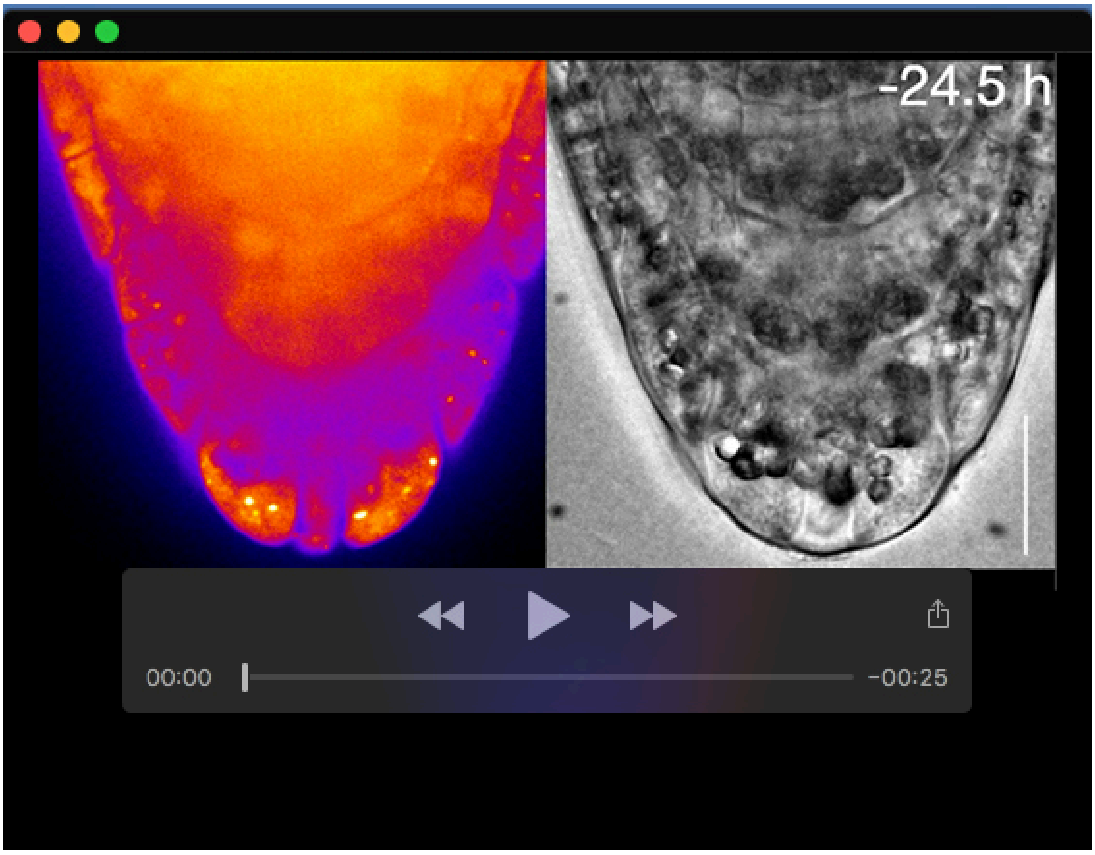

Movie 4. Time-lapse movie showing autophagosome formation in the outermost root cap cells visualized by *35Spro:GFP-ATG8a* Scale bar, 20 µm.

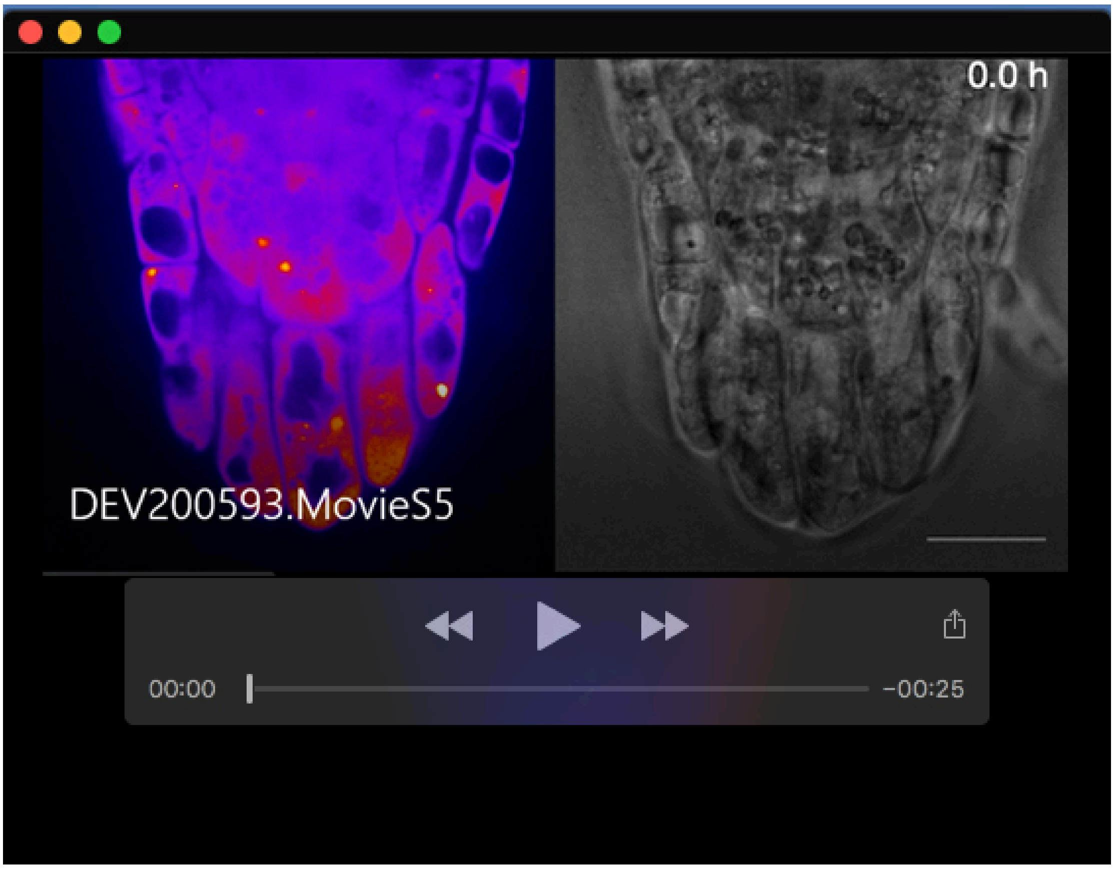

Movie 5. Time-lapse movie showing the absence of autophagosome formation in *atg5-1* root cap cells transgenically expressing *35Spro:GFP-ATG8a.*
Scale bar, 20 µm.

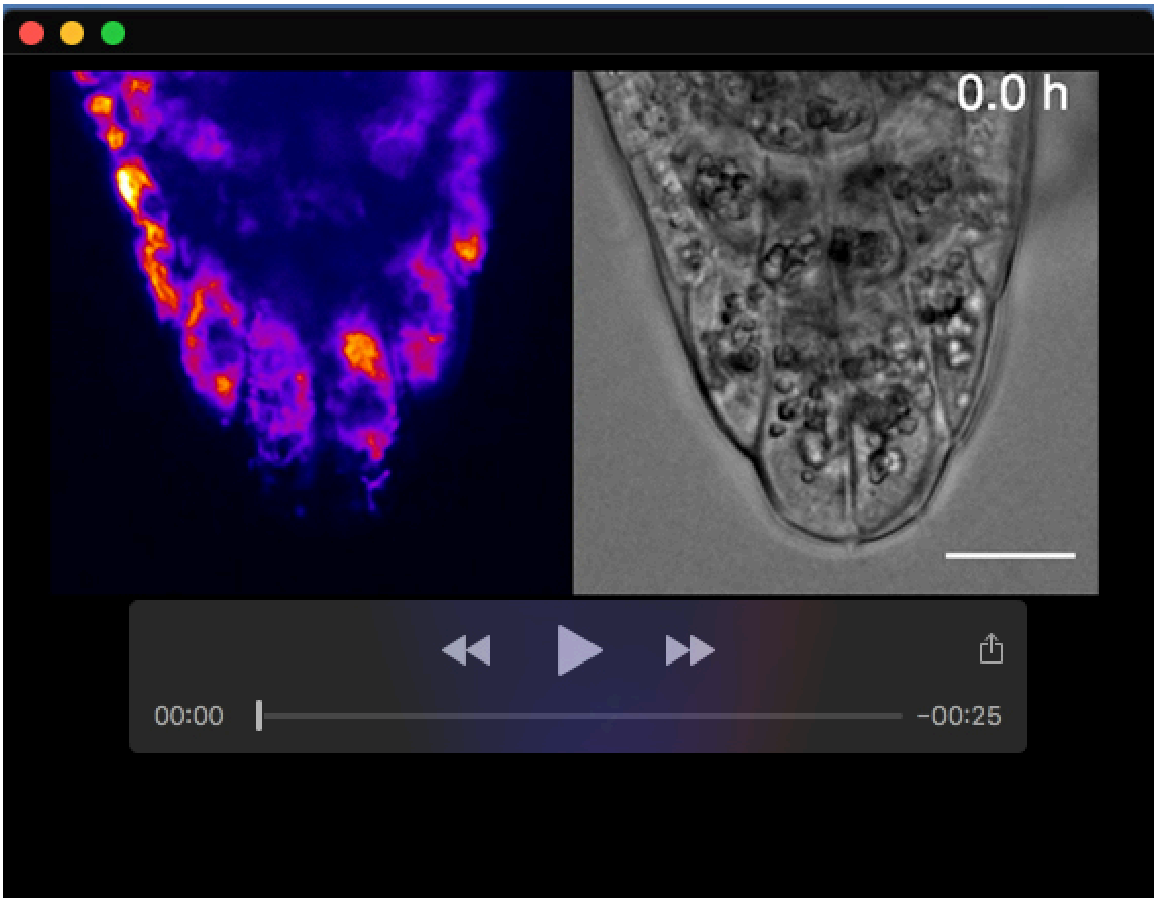

Movie S6. Time-lapse movie showing the morphological transition of vacuoles during cell detachment in *atg5-1* root cap cells Scale bar, 20 µm.

Development • Supplementary information

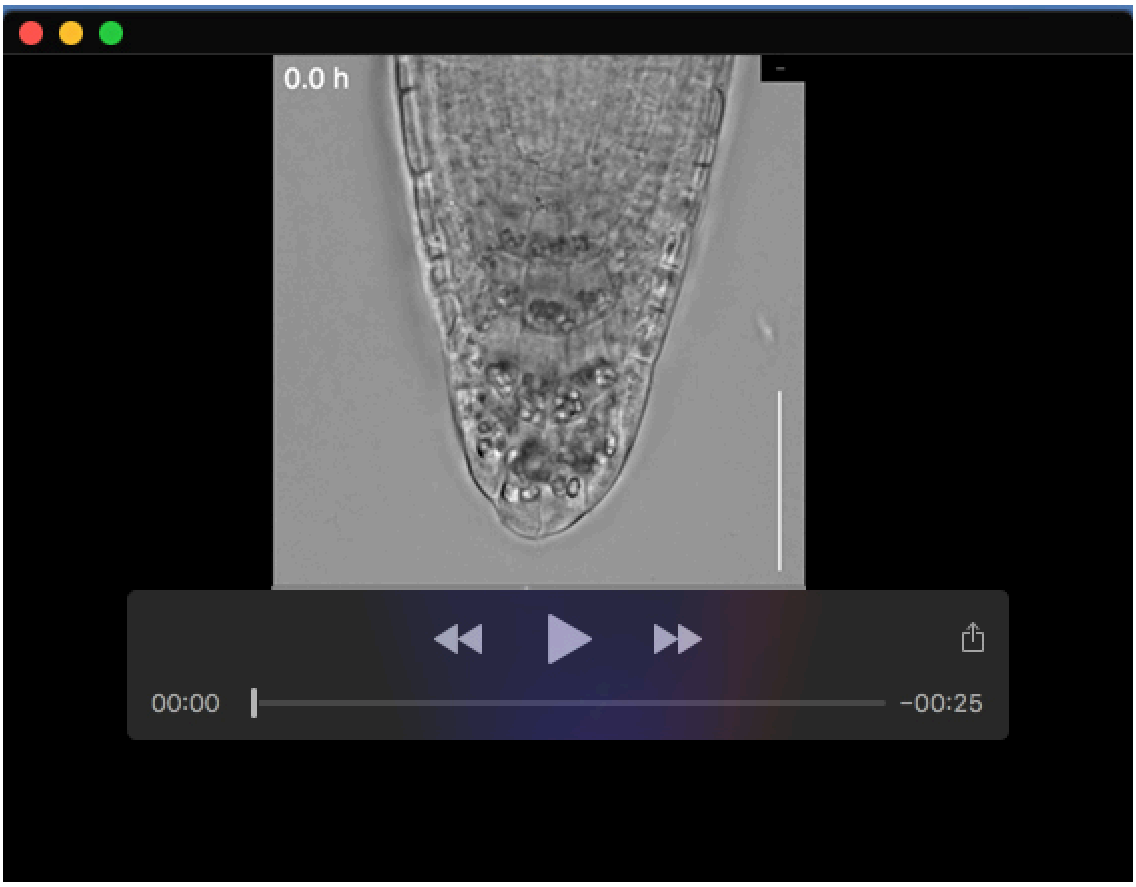

Movie 7. Time-lapse movie showing root cap cell detachment in wild-type plants
Scale bar, 50 µm.

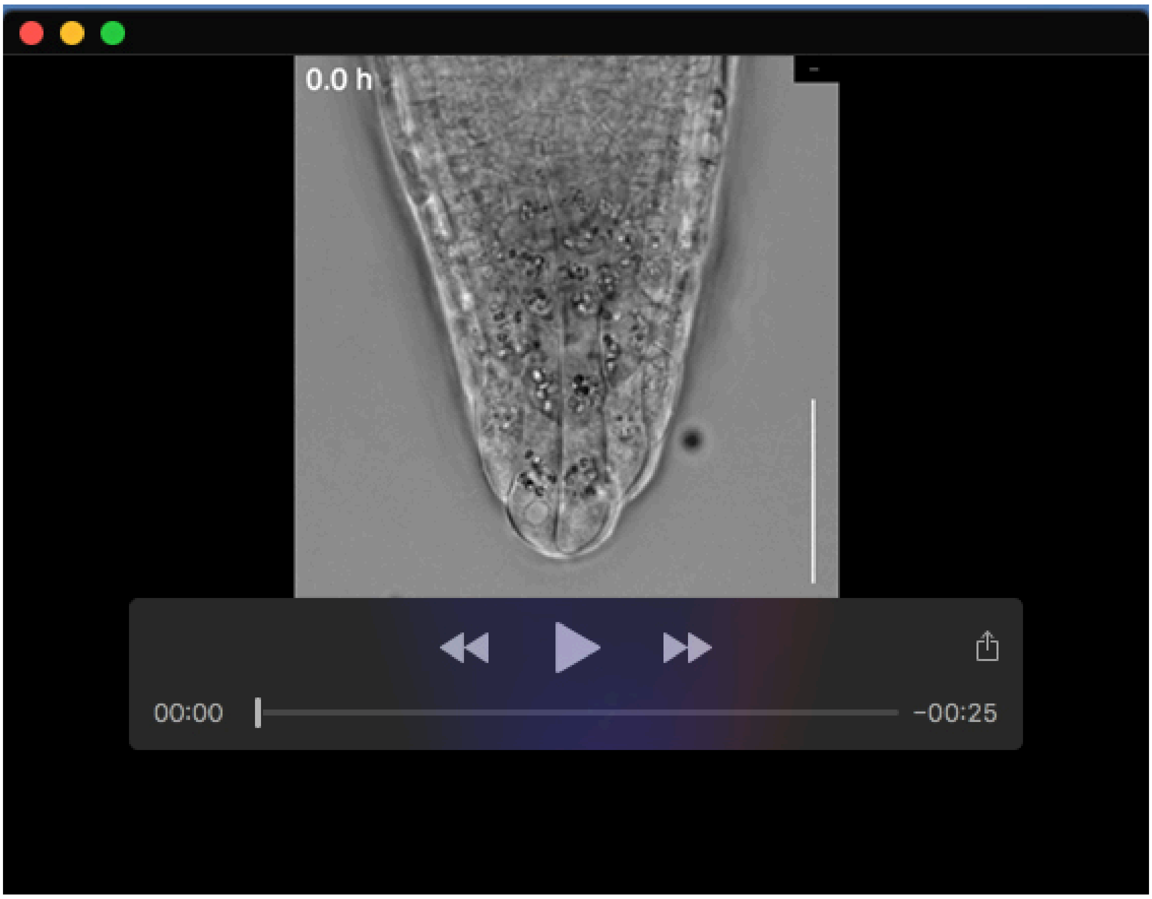

Movie 8. Time-lapse movie showing root cap cell detachment in *atg5-1* plants Scale bar, 50 µm.

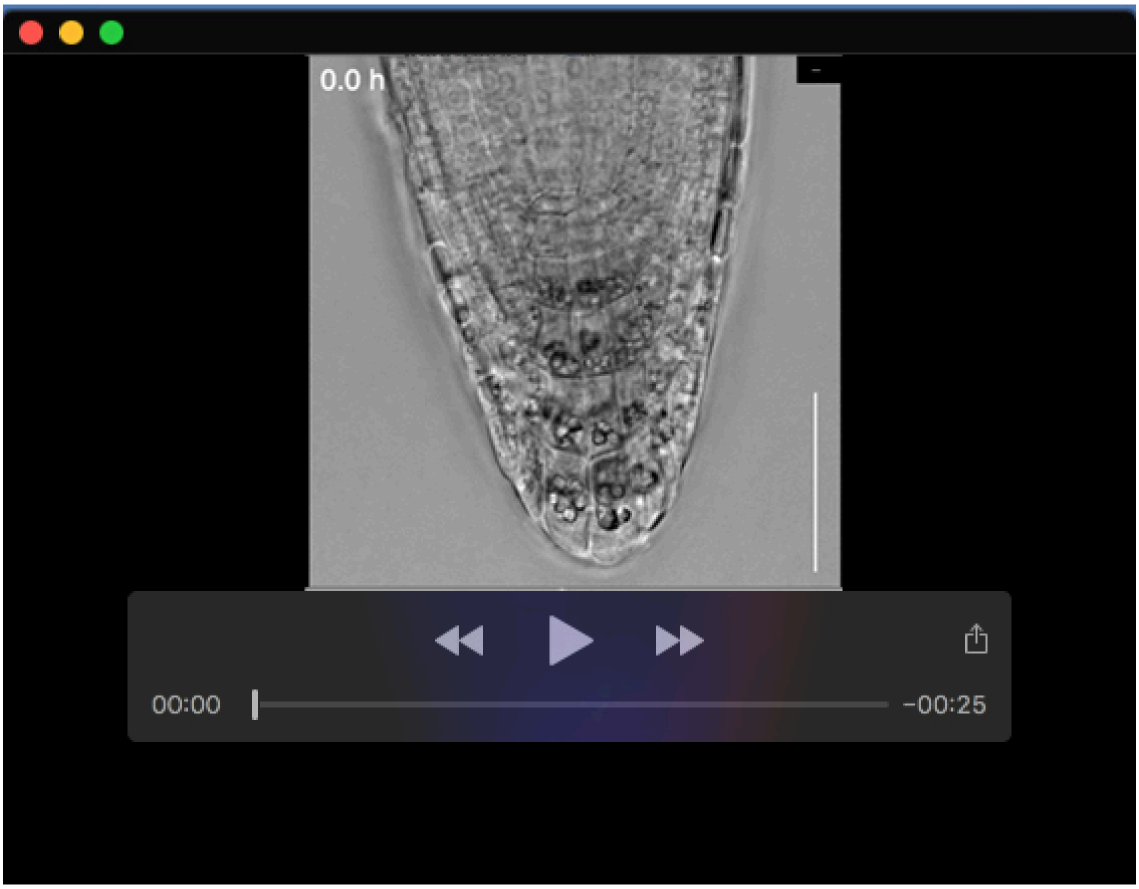

Movie 9. Time-lapse movie showing root cap cell detachment in *atg5-1* plants complemented with *ATG5pro:ATG5-GFP*
Scale bar, 50 µm.

Development • Supplementary information

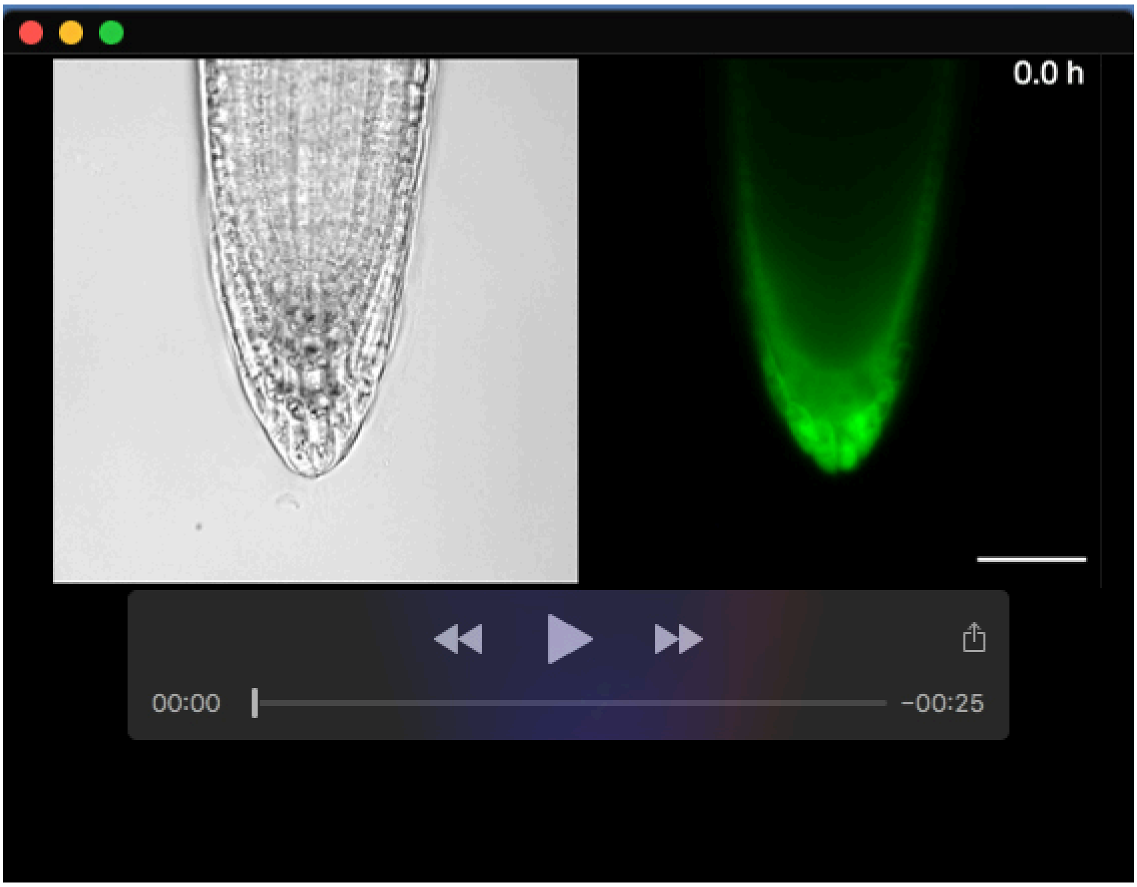

Movie 10. Time-lapse movie showing root cap cell detachment in *atg5-1* plants complemented with *BRN1pro:ATG5-GFP* Scale bar, 50 µm.

Development • Supplementary information

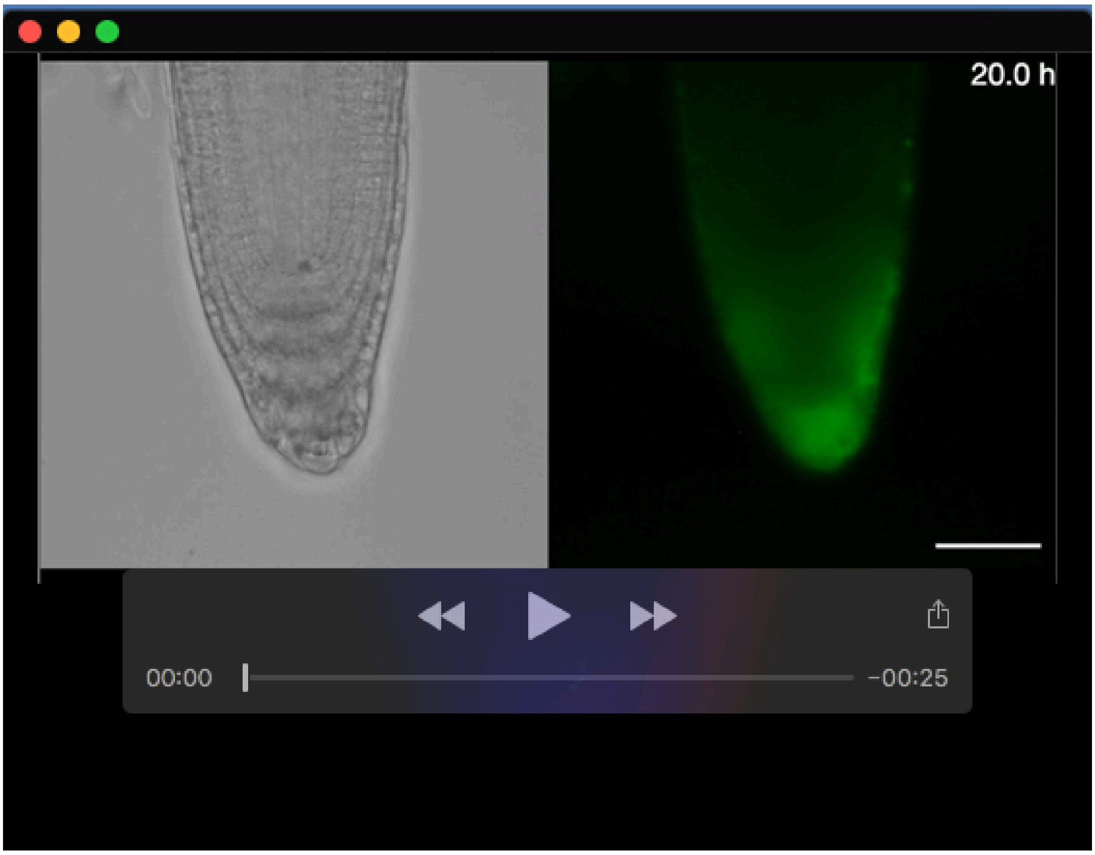

Movie 11. Time-lapse movie showing root cap cell detachment in *atg5-1* plants complemented with *RCPGpro:ATG5-GFP*

Scale bar, 50 µm.

Development • Supplementary information
