# Supplementary material for: Autophagy promotes organelle clearance and organized cell separation of living root cap cells in Arabidopsis thaliana
Source: Development. 2022 Jun 16;149(11):dev200593. doi: 10.1242/dev.200593 (PMC9245187; doi:10.1242/dev.200593)
Supplement: Reviewer comments [file develop-149-200593_review_history.pdf]

Autophagy promotes organelle clearance and organized cell separation of living root cap cells in *Arabidopsis thaliana*

Tatsuaki Goh, Kaoru Sakamoto, Pengfei Wang, Saki Kozono, Koki Ueno, Shunsuke Miyashima, Koichi Toyokura, Hidehiro Fukaki, Byung-Ho Kang and Keiji Nakajima
DOI: 10.1242/dev.200593

Editor: Yka Helariutta

Review timeline
Original submission: 12 February 2022
Editorial decision: 14 March 2022
First revision received: 30 March 2022
Accepted: 19 April 2022

Original submission

First decision letter

MS ID#: DEVELOP/2022/200593

MS TITLE: Autophagy promotes organelle clearance and organized cell separation of living root cap cells in *Arabidopsis thaliana*

AUTHORS: Tatsuaki Goh, Kaoru Sakamoto, Pengfei Wang, Saki Kozono, Koki Ueno, Shunsuke Miyashima, Koichi Toyokura, Hidehiro Fukaki, Byung-Ho Kang, and Keiji Nakajima

I have now received all the referees reports on the above manuscript, and have reached a decision. The referees' comments are appended below, or you can access them online: please go to BenchPress and click on the 'Manuscripts with Decisions' queue in the Author Area.

The overall evaluation is positive and we would like to publish a revised manuscript in Development, provided that the referees' comments can be satisfactorily addressed. Please attend to all of the reviewers' comments in your revised manuscript and detail them in your point-by-point response. If you do not agree with any of their criticisms or suggestions explain clearly why this is so.

We are aware that you may currently be unable to access the lab to undertake experimental revisions. If it would be helpful, we encourage you to contact us to discuss your revision in greater detail. Please send us a point-by-point response indicating where you are able to address concerns raised (either experimentally or by changes to the text) and where you will not be able to do so within the normal timeframe of a revision. We will then provide further guidance. Please also note that we are happy to extend revision timeframes as necessary.

Reviewer 1

Advance summary and potential significance to field

This manuscript uses high resolution microscopy to understand the spatio-temporal cellular developmental events that characterise the final maturation of the root cap cells in Arabidopsis thaliana, revealing the important role of autophagy in these processes. The manuscript provides

© 2022. Published by The Company of Biologists under the terms of the Creative Commons Attribution License (https://creativecommons.org/licenses/by/4.0/).

clear evidence of previously uncharacterised developmental mechanisms in the structural and functional maturation of the root cap.

Comments for the author

I think the data are convincing, and that the the manuscript is very well written and illustrated, and very 'publication-ready'. I only have one major point, as well as a few minor ones.

Major point:
1) Quantification Apart from Figure S1, there is no quantification of data anywhere in the manuscript. It is therefore possible that each figure is based on the observation of a single root, which would obviously be problematic. It is therefore important for the authors to be clear a) how many roots were examined for each separate experiment and b) what proportion of those roots showed the pattern illustrated in the figures. This should be stated in the figures themselves (e.g. '4/5'), and in the figure legends (e.g. data are representative of 5 roots, of which 4 showed the pattern observed here).

Minor points:
1) Figures Some of the figure panels are rather small and difficult to see at normal zoom particularly the bright-field images. I would suggest making Figure 1A and 1C larger (they might as well occupy the same width as Figure 1B), and perhaps re-arranging Figure 3 so that it is in 'portrait' as 3x6 grid (with time arranged vertically), rather than landscape as 6x3 grid. This should allow the images to be made larger.
2) Discussion The discussion feels quite long, and contains quite a lot of re-statement of the results, which is unnecessary. I think this could be condensed to make it easier to read and more 'punchy'.
3) Line 180
"The entire volume" - this is a bit of an exaggeration. The majority of the volume would be more accurate.
4) Line 233
"as ATG8" --> similar to ATG8
5) Line 254
"GFP-tagged GFP5" --> GFP-tagged ATG5
6) Line 276
"ATG-GFP" --> ATG5-GFP

Reviewer 2

Advance summary and potential significance to field

The manuscript of Goh et al. investigates the process of root cap shedding in Arabidopsis primary roots. The authors specifically describe the role that authophagy plays in this process. They find that autophagy is upregulated in the latest step of collumella differentiation, just before shedding, and demonstrate that it is important for organellar re-arrangements, especially formation of a central vacuole, as well as a coordinated execution of shedding of the collumella layer. In their experiments, the authors beautifully combine advanced microscopy allowing for long-term root cap tracking, with pertinent combinations of markers and mutants available in Arabidopsis. The CLEM analysis is also wonderful. Overall, this is a great study that I was very excited to read and I have only minor comments and suggestions for the authors that I hope might further improve their work.

Comments for the author

line 226: I am not a specialist on E64d action - but in how many cells in the Arabidopsis root does E64d not induce "autophagic body-like aggregates". If it is happening in all cells, then maybe this finding is not so relevant. It is not clear to me whether E64d induces autophagocytosis, or whether it simply reveals it?
There is very little in terms of confocal feature quantifications and statistics in this work. I am very reluctant to ask the authors for strict quantifications, because I am aware that quantifying differences in vacuolar morphologies (for example) between mutant and wt would be a nightmare. Still, it is hard for a non-specialist to appreciate the strict differences in vacuolar dynamics

© 2022. Published by The Company of Biologists under the terms of the Creative Commons Attribution License (https://creativecommons.org/licenses/by/4.0/).

between mutant and wt in the movies, for example. I imagine how hard it is to obtain such movies, but can the authors at least indicate, whether a given movie is unique, or whether it is a representative example of (n) movies?
Same of the for Figure 5A-D, where the differences between mutant and wt look very convincing. In the absence of quantifications, could the authors maybe simply provide some more examples in a Supplementary panel? In the same vein, I find Fig. 5E-L very convincing, for example, because of its redundancy, i.e. the pictures of different atg mutants showing similar FDA phenotypes. Maybe the authors could simply extend on that, showing a few more wt pictures, or at least indicating that this is a representative example of n+ observations?
Continuing with Fig. 5E-L, I found that not only FDA is different, but that shedding wt cells also show more PI uptake? Which would go nicely together with lack of FDA staining? Did the authors try to quantify this? It looks like the mutant show much less intracellular PI?
line 251 (Fig. S5C,D): I find this difference quite striking, nearly qualitative (again, if the authors do not want to quantify this, maybe provide more than one example?). I would suggest to put this result in the main Figures?

Reviewer 3

Advance summary and potential significance to field

Autophagy promotes organelle clearance and organized cell separation of living root cap cells in Arabidopsis thaliana by Goh et al.
The manuscript describes a topic that is catching a lot of attention nowadays which can be seen from the papers uploaded on BioRixv recently. Autophagy is an important topic, which can shed light on different processes. Here the authors focus on columella development, which is a nice system to gain more insight in this process. Even though there is strong overlap in results and ideas of a paper on BioRixv, I think the live cell imaging has a highly additive value to this work. I have several comments added to my report and with these major revisions I think the manuscript is acceptable for publication in Development.

Comments for the author

Comments:
Line 87 Bennet wrong reference, should be Kamiya et al Line 95 Do not contain large vesicles: The authors show in … that there are large amyloplasts and also various other papers show this.
Line 140 include what the growing conditions are.
Line 149 Bright-field….. these observations are not new, has been reported already several times before (include refs)
Fig 2A: What happens when a layer is detached, are all the organelles reorganized?
Include timepoints after detaching.
Fig 2A include close ups of outer cells, it is difficult to see the different aspects in the columella cells.
Fig 3 image quality is poor, just show the outer layers in zoom version.
Line 205/6; Fig 3B,C The confocal images of the 35SGFPATG8a are difficult to interpret, in the upper part of the root cap/ stem cell niche the staining is strong and doesn't correlate with autophagosomes and in the outer layer is connected? Can you better explain what we see and why?
In the study BRNs and SMB are used, but FEZ is not taken along, while this one has a different root cap with less layers and different behavior. Please inc

© 2022. Published by The Company of Biologists under the terms of the Creative Commons Attribution License (https://creativecommons.org/licenses/by/4.0/).

First revision

Author response to reviewers' comments

We appreciate the overall positive comments from the reviewers on our manuscript. We incorporated the comments and revised the manuscript accordingly. In the accompanying file named "Goh220331_track_changes.docx", revisions made to address the reviewer comments are highlighted in yellow. The manuscript is now checked by a professional English editor. Changes based on their suggestions are highlighted in gray.

Response to Reviewers

Reviewer 1
Reviewer 1 Advance Summary and Potential Significance to Field:
This manuscript uses high resolution microscopy to understand the spatio-temporal cellular developmental events that characterise the final maturation of the root cap cells in Arabidopsis thaliana, revealing the important role of autophagy in these processes. The manuscript provides clear evidence of previously uncharacterised developmental mechanisms in the structural and functional maturation of the root cap.

Reviewer 1 Comments for the Author:
I think the data are convincing, and that the manuscript is very well written and illustrated, and very 'publication-ready'. I only have one major point, as well as a few minor ones.

[Response]
We thank the reviewer for valuable and constructive comments on our manuscript. We revised the text and figures according to the reviewer's advice. Responses to the specific comments are described below.

Major point:
1) Quantification
Apart from Figure S1, there is no quantification of data anywhere in the manuscript. It is therefore possible that each figure is based on the observation of a single root, which would obviously be problematic. It is therefore important for the authors to be clear a) how many roots were examined for each separate experiment and b) what proportion of those roots showed the pattern illustrated in the figures. This should be stated in the figures themselves (e.g. '4/5'), and in the figure legends (e.g. data are representative of 5 roots, of which 4 showed the pattern observed here).

[Response]
We appreciate the reviewer's comments on this important issue. We observed three to five roots in each experiment and confirmed that they all showed comparable phenotypes. The images shown in each figure are representative. We added the number of the observed roots and stated that the images are representative in the figure legends (Figs. 2-7 and S2-S5). As all observations were reproducible (i.e. 100%), we would like not to label each image with the numbers "x/x", but rather state it in the figure legends.

Minor points:
1) Figures
Some of the figure panels are rather small and difficult to see at normal zoom, particularly the bright-field images. I would suggest making Figure 1A and 1C larger (they might as well occupy the same width as Figure 1B), and perhaps re-arranging Figure 3 so that it is in 'portrait' as 3x6 grid (with time arranged vertically), rather than landscape as 6x3 grid. This should allow the images to be made larger.

[Response]
(We interpret Fig. 1 in the comment as Fig. 2) In response to the reviewer's suggestion, we added magnified images of the outer root cap layers of the bright-field images in Fig. 2A (bottom row). Fig. 2C was enlarged to have the same width as 2B.
As for Fig. 3, we would like to keep the landscape style (6x3 grid), because rearranging them to a portrait style (3x6 grid) would be rather counterintuitive to see the time flow, and would have

© 2022. Published by The Company of Biologists under the terms of the Creative Commons Attribution License (https://creativecommons.org/licenses/by/4.0/).

different arrangement from those of other figures. As an alternative way to achieve better visualization, we inserted magnified views of the bright-field images (new raw B).

2) Discussion
The discussion feels quite long, and contains quite a lot of re-statement of the results, which is unnecessary. I think this could be condensed to make it easier to read and more 'punchy'.

[Response]
We thank the reviewer and fully agree with this point. We removed re-statement of the results as much as possible. The total length of the discussion was shortened by 30 lines in the manuscript.

3) Line 180
"The entire volume" - this is a bit of an exaggeration. The majority of the volume would be more accurate.

[Response]
We changed "the entire volume" to "most of the (cell) volume" (lines 178, 230 and 768 [in the legend of Supplemental Fig S2]).

4) Line 233
"as ATG8" --> similar to ATG8

[Response]
We changed *"as ATG8"* to *"similar to ATG8"* (line 224).

5) Line 254
"GFP-tagged GFP5" --> GFP-tagged ATG5

[Response]
We corrected "GFP-tagged GFP5" to "GFP-tagged ATG5" (line 245).

6) Line 276
"ATG-GFP" --> ATG5-GFP

[Response]
We corrected "ATG-GFP" to "ATG5-GFP" (line 267, Movies S9, S10 titles).

Reviewer 2
Reviewer 2 Advance Summary and Potential Significance to Field:
The manuscript of Goh et al. investigates the process of root cap shedding in Arabidopsis primary roots. The authors specifically describe the role that authophagy plays in this process. They find that autophagy is upregulated in the latest step of collumella differentiation, just before shedding, and demonstrate that it is important for organellar re-arrangements, especially formation of a central vacuole, as well as a coordinated execution of shedding of the collumella layer. In their experiments, the authors beautifully combine advanced microscopy allowing for long-term root cap tracking, with pertinent combinations of markers and mutants available in Arabidopsis. The CLEM analysis is also wonderful. Overall, this is a great study that I was very excited to read and I have only minor comments and suggestions for the authors that I hope might further improve their work.

[Response]
We thank the reviewer for valuable and constructive comments on our manuscript. We revised the text and figures according to the reviewer's advice. Responses to the specific comments are as follows.

Reviewer 2 Comments for the Author:
1) *line 226: I am not a specialist on E64d action - but in how many cells in the Arabidopsis root does E64d not induce "autophagic body-like aggregates". If it is happening in all cells, then maybe this finding is not so relevant. It is not clear to me whether E64d induces autophagocytosis,*

© 2022. Published by The Company of Biologists under the terms of the Creative Commons Attribution License (https://creativecommons.org/licenses/by/4.0/).

or whether it simply reveals it?

[Response]
We appreciate this important comment. Subcellular components delivered to the vacuoles by autophagosomes are immediately degraded by lytic enzymes in the vacuolar lumen. E- 64d is a cell-permeable cysteine protease inhibitor for many proteases including those in the vacuolar lumen. While the E-64d treatment was expected to visualize accumulation of components that are normally to be degraded in the vacuoles, especially in cells that have higher autophagic potentials, the effect of E-64d is not specific to autophagy as the reviewer pointed out. Indeed, the accumulation of the aggregates was more pronounced in the outermost layers of the root cap, but was also observed in other cells to some extent. Considering that the critical role of autophagy is more explicitly visualized in our genetic analysis, we now removed the E-64d data from Fig. S5 (previous Fig. S3), and its description from the text. The order of the figures has been changed to suit these changes.

2) *There is very little in terms of confocal feature quantifications and statistics in this work. I am very reluctant to ask the authors for strict quantifications, because I am aware that quantifying differences in vacuolar morphologies (for example) between mutant and wt would be a nightmare. Still, it is hard for a non-specialist to appreciate the strict differences in vacuolar dynamics between mutant and wt in the movies, for example. I imagine how hard it is to obtain such movies, but can the authors at least indicate, whether a given movie is unique, or whether it is a representative example of (n) movies?*

[Response]
We appreciate the reviewer for raising this important issue. A related comment was also given by Reviewer 1. We observed three to five roots in each experiment and confirmed that the observed phenotypes are fully reproducible. We added information as to the number of samples in the figure legends (Figs. 2-7 and S2-S5). We also thank the reviewer for acknowledging the difficulty of quantifying the vacuolar dynamics.

Same of the for Figure 5A-D, where the differences between mutant and wt look very convincing. In the absence of quantifications, could the authors maybe simply provide some more examples in a Supplementary panel? In the same vein, I find Fig. 5E-L very convincing, for example, because of its redundancy, i.e. the pictures of different atg mutants showing similar FDA phenotypes. Maybe the authors could simply extend on that, showing a few more wt pictures, or at least indicating that this is a representative example of n+ observations?

[Response]
We appreciate this comment, which is also related to the one by Reviewer 1. We observed three to five roots in each experiment and confirmed the reproducibility in all cases. We added the number of observed roots in the legend to Fig. 5.

Continuing with Fig. 5E-L, I found that not only FDA is different, but that shedding wt cells also show more PI uptake? Which would go nicely together with lack of FDA staining? Did the authors try to quantify this? It looks like the mutant show much less intracellular PI?

[Response]
We thank the reviewer for this comment. While it is possible that highly-vacuolated WT cells are more fragile than the cytosol-rich cells of autophagy-deficient mutants, cells densely stained with PI were also observed in the autophagy-deficient mutants, though the number of such cells varied with samples. We speculated that the variable staining might be attributable to the elapsed time after the cell detachment.

line 251 (Fig. S5C,D): I find this difference quite striking, nearly qualitative (again, if the authors do not want to quantify this, maybe provide more than one example?). I would suggest to put this result in the main Figures?

[Response]
In response to the reviewer's comment, we moved the *BRN1pro:GUS-GFP* lines (former Fig. S5C,D) to the main Fig. 5 (Fig. 5M,N). This result was reproducible in all three roots

© 2022. Published by The Company of Biologists under the terms of the Creative Commons Attribution License (https://creativecommons.org/licenses/by/4.0/).

examined for each genotype (now mentioned in the legend to Fig. 5).

Reviewer 3

Reviewer 3 Advance Summary and Potential Significance to Field:

Autophagy promotes organelle clearance and organized cell separation of living root cap cells in Arabidopsis thaliana by Goh et al. The manuscript describes a topic that is catching a lot of attention nowadays which can be seen from the papers uploaded on BioRixv recently. Autophagy is an important topic, which can shed light on different processes. Here the authors focus on columella development, which is a nice system to gain more insight in this process. Even though there is strong overlap in results and ideas of a paper on BioRixv, I think the live cell imaging has a highly additive value to this work. I have several comments added to my report and with these major revisions I think the manuscript is acceptable for publication in Development.

[Response]
We thank the reviewer for the valuable and constructive comments on our manuscript. We revised the text and figures according to the reviewer's advice. Responses to the specific comments are as follows.

Reviewer 3 Comments for the Author:

Comments:

Line 87 Bennet wrong reference, should be Kamiya et al

[Response]
We removed Bennett et al., 2010 (line 85).

Line 95 Do not contain large vesicles: The authors show in … that there are large amyloplasts and also various other papers show this.

[Response]
We interpret this comment as pointing out the discrepancy between the statement of the absence of large amyloplasts in the outermost cells in the Introduction and our observation showing the presence of fairly large amyloplasts in these cells immediately after the detachment of the abutting outer cell layer (Fig. 2). We consider this discrepancy to have arisen from our use of the time-lapse imaging that could capture the narrow time window where the amyloplasts are detectable before degradation. To clear this point, we changed this sentence from "In contrast, columella cells constituting the outermost root cap layer do not contain large amyloplasts" to "By contrast, fully maturated columella cells at the outermost root cap layer do not contain large amyloplast" (lines 92-93).

Line 140 include what the growing conditions are.
[Response]
In response to this comment, we added more detailed descriptions of growth conditions to the Results and Methods sections (lines 137-140 and 439-443).

Line 149 Bright-field….. these observations are not new, has been reported already several times before (include refs)

[Response]
In response to this comment, we added two references describing the root cap detachment processes (Fendrych et al., 2014; Shi et al., 2018) (line 150), and changed “revealed” to “confirmed” (line 148).

Fig 2A: What happens when a layer is detached, are all the organelles reorganized? Include timepoints after detaching.

[Response]
We appreciate this important question. The detached cells remained fully vacuolated. We added an image after completion of the cell separation (22.5 h) to Fig. 2A, and revised the

© 2022. Published by The Company of Biologists under the terms of the Creative Commons Attribution License (https://creativecommons.org/licenses/by/4.0/).

corresponding text accordingly (lines 161-162).

Fig 2A include close ups of outer cells, it is difficult to see the different aspects in the columella cells.

[Response]
We added magnified views of the bright-field images in Fig. 2A (bottom row). The same comment was also given by Reviewer 1.

Fig 3 image quality is poor, just show the outer layers in zoom version.

[Response]
We added magnified views of the bright-field images in Fig. 3 (new raw B). The same comment was also given by Reviewer 1.

Line 205/6; Fig 3B,C The confocal images of the 35SGFPATG8a are difficult to interpret, in the upper part of the root cap/ stem cell niche the staining is strong and doesn't correlate with autophagosomes and in the outer layer is connected? Can you better explain what we see and why?

[Response]
We appreciate this question. Upon autophagy activation, ATG8a relocates from cytosol to autophagosome membranes via conjugation with phosphatidylethanolamine. Formation of the ATG8-labelled punctate structures are thus considered to correlated with autophagic activity in this marker line (Yoshimoto et al., 2004, Plant Cell). We found that the number of GFP-ATG8a-labelled punctate structures increased at the specific timing in the outermost root cap cells (Fig. 3D, white arrowheads), whereas the GFP fluorescence was uniformly distributed to the cytosol in the inner layers. The strong signal intensity in the stem cell region is likely due to the overexpression of GFP-ATG8 by the *35S* promoter and cytoplasm-rich nature of these cells.

In the study BRNs and SMB are used, but FEZ is not taken along, while this one has a different root cap with less layers and different behavior. Please inc

[Response]
In this manuscript, we focused on maturation and separation of the outer root cap cells. Previous studies have shown that *SMB*, *BRN1* and *BRN2* regulate these processes with partially overlapping functions (Willemsen et al., 2008; Bennett et al., 2010; Fendrych et al., 2014). We have reported that *BRN1* and *BRN2* are expressed specifically in the outer root cap layers and promote cell separation via direct activation of a genes encoding a cell wall modifying enzyme RCPG (Kamiya et al., 2016). Accordingly, we here used the *BRN1* and *RCPG* promoters for complementation. Along the same line, we discussed a possibility that BRNs activate autophagy in the outer root cap cells. We agreed that FEZ is another key transcription factor of root cap development, but to our knowledge, *FEZ* acts in the inner root cap layers to regulate the stem cell division, and thus unlikely to act in the maturation and autophagy activation in the outer root cap cells.

© 2022. Published by The Company of Biologists under the terms of the Creative Commons Attribution License (https://creativecommons.org/licenses/by/4.0/).

Second decision letter

MS ID#: DEVELOP/2022/200593

MS TITLE: Autophagy promotes organelle clearance and organized cell separation of living root cap cells in *Arabidopsis thaliana*

AUTHORS: Tatsuaki Goh, Kaoru Sakamoto, Pengfei Wang, Saki Kozono, Koki Ueno, Shunsuke Miyashima, Koichi Toyokura, Hidehiro Fukaki, Byung-Ho Kang, and Keiji Nakajima
ARTICLE TYPE: Research Article
I am happy to tell you that your manuscript has been accepted for publication in Development, pending our standard ethics checks.

Reviewer 1

Advance summary and potential significance to field

I didn't have many comments about this manuscript, and the authors have satisfactorily addressed all the comments I made. I have no further comments to make, other than to re-iterate that this is a very nice story!

Comments for the author

N/A

Reviewer 2

Advance summary and potential significance to field

As can be seen from my initial review, I was already very happy with the manuscript and had only minor comments. I have now read the authors reply and I can confirm that they have adequately adressed my remaining concerns.

Comments for the author

As can be seen from my initial review, I was already very happy with the manuscript and had only minor comments. I have now read the authors reply and I can confirm that they have adequately adressed my remaining concerns.

Reviewer 3

Advance summary and potential significance to field

Autophagy promotes organelle clearance and organized cell separation of living root cap cells in Arabidopsis thaliana by Goh et al. The manuscript describes a topic that is catching a lot of attention nowadays which can be seen from the papers uploaded on BioRixv recently. Autophagy is an important topic, which can shed light on different processes. Here the authors focus on columella development, which is a nice system to gain more insight in this process. Even though there is strong overlap in results and ideas of a paper on BioRixv, I think the live cell imaging has a highly additive value to this work.

Comments for the author

I think the authors have addressed my concerns and accept the manuscript for publication.

© 2022. Published by The Company of Biologists under the terms of the Creative Commons Attribution License (https://creativecommons.org/licenses/by/4.0/).
